# A Bayesian method for detecting pairwise associations in compositional data

**DOI:** 10.1371/journal.pcbi.1005852

**Published:** 2017-11-15

**Authors:** Emma Schwager, Himel Mallick, Steffen Ventz, Curtis Huttenhower

**Affiliations:** 1 Department of Biostatistics, Harvard T.H. Chan School of Public Health, Boston, Massachusetts, United States of America; 2 Broad Institute of MIT and Harvard, Cambridge, Massachusetts, United States of America; 3 Department of Computer Science and Statistics, University of Rhode Island, Kingstown, Rhode Island, United States of America; 4 Department of Biostatistics and Computational Biology, Dana-Farber Cancer Institute, Boston, Massachusetts, United States of America; University of Minnesota, UNITED STATES

## Abstract

Compositional data consist of vectors of proportions normalized to a constant sum from a basis of unobserved counts. The sum constraint makes inference on correlations between unconstrained features challenging due to the information loss from normalization. However, such correlations are of long-standing interest in fields including ecology. We propose a novel Bayesian framework (BAnOCC: Bayesian Analysis of Compositional Covariance) to estimate a sparse precision matrix through a LASSO prior. The resulting posterior, generated by MCMC sampling, allows uncertainty quantification of any function of the precision matrix, including the correlation matrix. We also use a first-order Taylor expansion to approximate the transformation from the unobserved counts to the composition in order to investigate what characteristics of the unobserved counts can make the correlations more or less difficult to infer. On simulated datasets, we show that BAnOCC infers the true network as well as previous methods while offering the advantage of posterior inference. Larger and more realistic simulated datasets further showed that BAnOCC performs well as measured by type I and type II error rates. Finally, we apply BAnOCC to a microbial ecology dataset from the Human Microbiome Project, which in addition to reproducing established ecological results revealed unique, competition-based roles for Proteobacteria in multiple distinct habitats.

## Author summary

Data from many fields are available primarily in the form of proportions, also referred to as compositions, which impose mathematical constraints on identifying interactions among components in the underlying systems. In particular, correlations cannot be calculated directly from proportions or from count data that give rise to them. Methods that work around this difficulty generally do so by imposing strong assumptions about the distribution of underlying data or associated correlations, and these in turn often prevent quantifying uncertainty in the resulting estimates of correlation. We developed a statistical model (BAnOCC: Bayesian Analysis of Compositional Covariance) that both estimates correlations between counts or proportions and provides a posterior distribution for each correlation that quantifies how uncertain the estimate is. BAnOCC does well at controlling the number of false positives in simulated data and can be practically applied to a wide range of proportional data types.

## Introduction

A long-standing goal of applied statistics in many fields has been identifying features associated significantly by a measure such as correlation [1,2]. When the features to be associated form a composition, inference of the correlation matrix is subject to the well-known problem of spurious correlation [3–6]. Compositional data in particular are vectors of proportions that sum to a fixed constant (typically one); they are usually thought of as the result of sum-normalizing an unobserved (or unrecorded) and unconstrained basis, following the terminology of [6]. The resulting sum-constraint of the compositional data means that any pairwise correlation measured using such data can be non-zero even if all the pairwise correlations on the unobserved count scale are zero, a phenomenon called spurious correlation [3]. The fact that all the features sum to one also makes the correlation matrix on the unobserved counts (that is, the basis correlation matrix) non-identifiable without untestable, though perhaps not unreasonable, assumptions [7–10]. Any method thus offers at best a partial reconstruction of the unobserved count correlation matrix, and the interest in characterizing such correlations in fields from geology to ecology has led to a variety of approaches.

In the context of microbial ecology, several methods have been proposed to identify significant ecological relationships from compositions; virtually all rely on some form of sparsity assumption and infer quantities relating to the log-transformed unobserved counts (hereafter referred to as the log-basis). The only technique that does not rely on a sparsity assumption is ReBoot [7], which estimates a “compositionally-corrected” correlation matrix using a permutation-based method. Friedman and Alm [8] proposed SparCC, which estimates the log-basis correlation matrix under the assumption that the correlations are on average small in magnitude. Fang et al. [9] noted that the resulting estimate is not guaranteed to be positive definite or that the elements will lie inside [–1, 1] and proposed CCLasso to estimate the log-basis correlation matrix using a LASSO penalty on the off-diagonal elements of the variance-covariance matrix. Ban et al. [10] similarly proposed REBACCA to estimate the log-basis correlation matrix; they use the same LASSO penalty function but a different likelihood function. Kurtz et al. [11] proposed SPIEC-EASI to estimate the log-basis precision matrix when the number of features is large by using sparse graph estimation techniques.

These approaches have difficulty quantifying uncertainty in the estimates, cannot incorporate uncertainty from the choice of tuning parameter, and are not flexible in the quantities they estimate. Friedman and Alm [8] proposed an inferential procedure based on the bootstrap, but offered no theoretical justification. Fang et al. [9] and Kurtz et al. [11] focused solely on estimation, while Ban et al. [10] used a subsampling method from Shah and Samworth [12] to stabilize the selection error rate. The LASSO-based methods [9–11] typically choose a shrinkage parameter and subsequently infer the log-basis covariance or precision matrix. Friedman and Alm [8], Fang et al. [9], and Ban et al. [10] all use the log-basis covariance matrix for network construction, while Kurtz et al. [11] use the log-basis precision matrix. This means that investigators typically must choose whether a precision or correlation matrix is best, and often use the resulting estimate with little guidance as to its uncertainty.

We address these issues by providing a flexible, fully Bayesian approach to identify correlations in compositional data. It is able to quantify uncertainty through the associated posterior and estimates both the log-basis correlation and precision matrix by modeling the composition directly. The graphical LASSO prior of [13] is used to estimate a sparse log-basis precision matrix (and hence a sparse log-basis correlation matrix) through a LASSO penalty, mitigating the non-identifiable nature of the unobserved count correlation matrix. We have implemented the resulting method as BAnOCC (Bayesian Analysis of Compositional Covariance). In this study, we also use a first-order Taylor expansion to approximate the compositional covariance as a function of the mean and variance of the unobserved counts. While not necessary to the development of our method, this expansion helps us explore the situations in which a naïve approach (ignoring the sum-constraint) might work. This approximation shows not only that the spurious correlation between two features can take any value in [−1,1] even if none of the features are correlated on the unobserved count scale, but also that both the variances and means of the unobserved counts control the magnitude and direction of the spurious correlation. Thus, we provide a novel characterization of the surprisingly broad circumstances under which compositionality can impede straightforward identification of the correlation matrix, and we provide the BAnOCC model to overcome this in datasets where it is possible.

## Methods

### Per-subject basis (unobserved and unconstrained count) and composition notation

The model assumes that a single subject’s composition, **C**_*i*_ = (*C*_*i*,1_,…,*C*_*i*,*p*_)^*T*^, is generated by the normalization of that subject’s unobserved and unconstrained counts, **X**_*i*_ = (*X*_*i*,1_,…,*X*_*i*,*p*_)^*T*^. That is, $C_{i}=\frac{X_{i}}{\overset{p}{\underset{j=1}{\sum }}X_{i,j}}$. We also assume that the unobserved counts for all subjects are independent and identically distributed (iid); this implies that the compositions are iid as well because the transformation is per-subject.

### Feature correlations and covariances in composition and unobserved counts

We also introduce notation for the covariance and correlation among the features. The covariance matrix of the unobserved counts is denoted by **Σ**_*X*_ = [*σ*_*X*,*jk*_], to be inferred from **C**_1_,…,**C**_*n*_. Similarly, the covariance matrix of the composition is denoted by **Σ**_*C*_ = [*σ*_*C*,*jk*_]. To construct the network of feature interactions, the relevant null hypotheses (one for each feature pair *j* and *k*) are that features *j* and *k* have a covariance of zero (*σ*_*X*,*jk*_ = 0); this is equivalent to testing if they are uncorrelated (*ρ*_*X*,*jk*_ = 0). We then define the unobserved count and compositional correlation matrices as **R**_*X*_ = [*ρ*_*X*,*jk*_] and **R**_*C*_ = [*ρ*_*C*,*jk*_], respectively.

## BAnOCC: Bayesian analysis of compositional covariance

**Fig 1:**
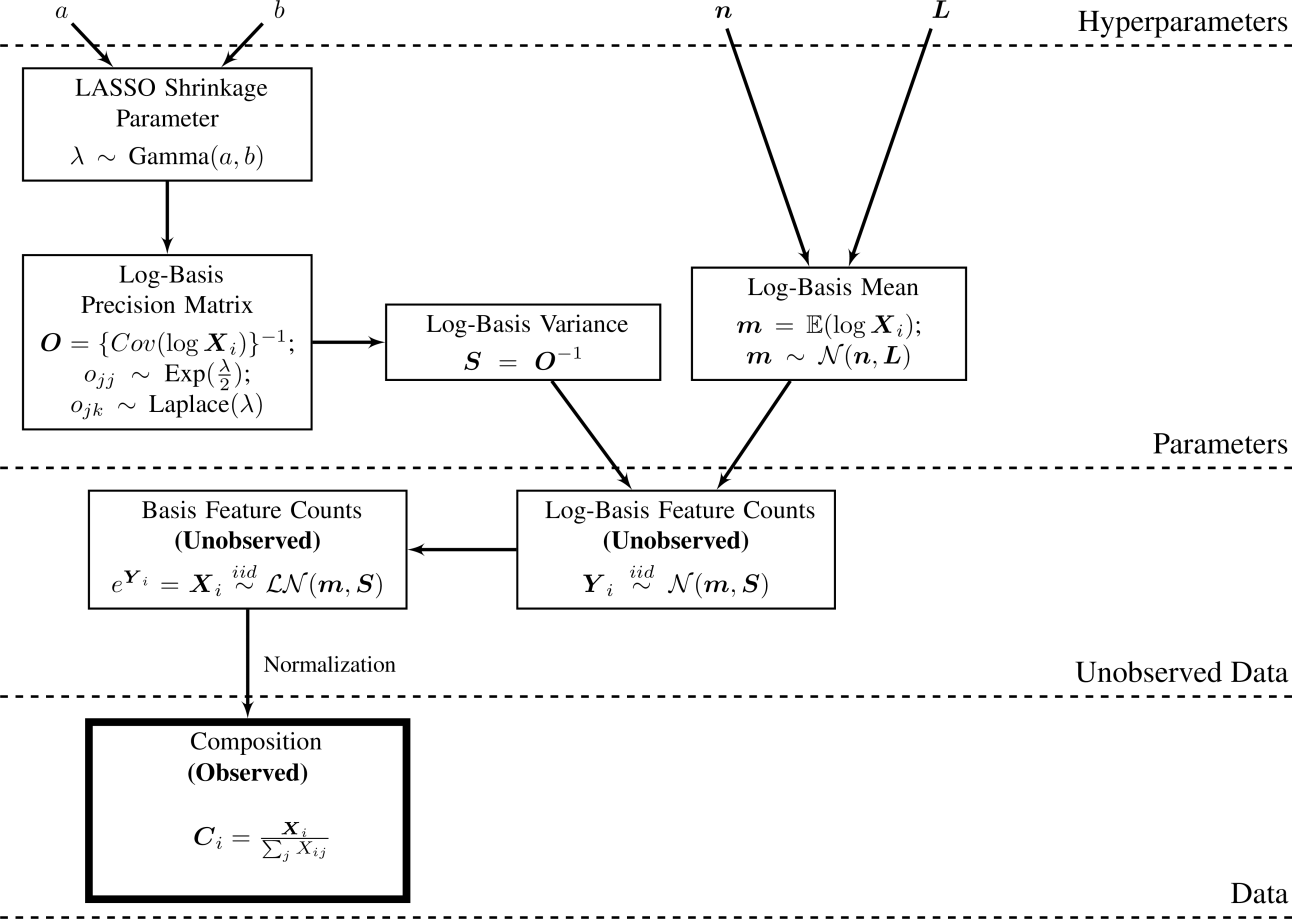
BAnOCC infers log-basis correlation and precision matrices from compositions by modeling unobserved and unconstrained counts. In the BAnOCC model, the observed compositions, **C**_i_, are derived by normalizing the unobserved counts **X**_i_. The BAnOCC model assumes that the **X**_i_ follow a log-normal distribution, parametrized by the log-basis mean **m** and covariance **S**. It places a normal prior on **m,** the GLASSO prior of [13] on the log-basis precision matrix **O,** and a hyperprior on the GLASSO shrinkage parameter λ (see Methods).

BAnOCC assumes that the unobserved counts follow a log-normal distribution and that their correlation matrix is sparse; it is parametrized with the log-basis precision matrix and the log-basis mean (**Fig 1**). Posteriors for the parameters of the model (and thus functions of them which are of interest) are inferred using MCMC sampling. This fully Bayesian treatment of the problem gives several advantages: a full posterior distribution to quantify the uncertainty in the estimates, the ability to place a prior on the sparsity parameter, and estimates of any function of the log-basis precision matrix, including the log-basis covariance and correlation matrices.

BAnOCC models the unobserved and unconstrained counts using a log-normal distribution with parameters based on the moments of the log-basis: $X_{i}\overset{iid}{∼}LN(m,S)$, such that m=E(log{X}) and **S** = Var(log{**X**}). This continuous approximation of the underlying unobserved count data is expected to perform well when the underlying counts have a large dynamic range. In ecology, for example, the log-normal distribution is used to model the (discrete) abundance across species [14,15]. In microbial ecology specifically, the logistic normal is sometimes assumed to be the generating distribution of the composition [10,11]; further, the (discrete) read counts are often simulated using a log-normal distribution [16,17]. The log-normal distribution also allows the totals to be easily integrated out of the likelihood.

### Parametrization of the likelihood

The likelihood is parametrized by the log-basis precision matrix **O** = **S**^−1^ and the log-basis mean **m**, and other parameters of interest like the log-basis covariance matrix **S** are sampled as transformations of these. By parametrizing using **O**, we are able to leverage a graphical LASSO prior to enforce sparsity on **O** and by extension **S**. Conveniently, the assumption of the log-normal distribution obviates the need to sample the covariance of the unobserved counts to determine the existence and direction of an association between two features on the unobserved count scale. This results because when some element of **S**, *s*_*jk*_, is zero, then the corresponding element of $\Sigma_{X},\sigma_{X,jk}\propto e^{s_{jk}}-1$ will also be zero; further, the non-zero elements of **S** and **Σ**_*X*_ will have the same sign (though not the same magnitude).

Under the log-normal assumption, the complete likelihood of the observed composition **c**_*i*_ and the latent total $t_{i}=\overset{p}{\underset{j=1}{\sum }}x_{i,j}$ is given by

$$L(m,O|c_{i},t_{i})=\frac{\exp[-\frac{1}{2}{\left\{log(c_{i}t_{i})-m\right\}}^{T}O\{log(c_{i}t_{i})-m\}]}{(t_{i}){\left(2\pi \right)}^{p/2}{\left|O\right|}^{-1/2}\prod_{j=1}^{p}c_{i,j}},$$ (1)

where $c_{i}=(c_{i,1},\ldots ,c_{i,p-1},1-\overset{p-1}{\underset{j=1}{\sum }}c_{i,j})$. A detailed derivation can be found in **S1 Text**. Fitting this likelihood directly is computationally expensive, as the presence of the latent totals necessitates exploring a space whose dimension depends on both *n* and *p*. However, (1) factors into two portions: a part dependent on the compositions **c**_*i*_, and the kernel of a log-normal distribution for the totals $t_{i}=\overset{p}{\underset{j=1}{\sum }}x_{i,j}$ with parameters $m_{i}^{*}=1^{T}O(m-log\{c_{i}\}){s^{2}}^{*}$ and ${s^{2}}^{*}=\frac{1}{1^{T}O1}$ (where **1** is a vector of 1’s). Integrating over the totals in (1) (**S1 Text**) gives the more computationally tractable marginal likelihood

$$L(m,O|c_{i})={\left|O\right|}^{1/2}\frac{\exp\{-\frac{1}{2}{\left(m-\log c_{i}\right)}^{T}O(m-{\log c}_{i})-\frac{{\left(m_{*}^{i}\right)}^{2}}{S^{2*}}\}{\left(2\pi \right)}^{1/2}{\left(s^{2*}\right)}^{1/2}}{{\left(2\pi \right)}^{p/2}\Pi_{j=1}^{p}c_{ij}}.$$

### Prior distributions

In order to mitigate the non-identifiability of the precision matrix **O,** BAnOCC uses a shrinkage prior to conservatively estimate the sparsest **O** consistent with the observed relative abundance data. This is the graphical LASSO prior of [13]:

$$p(O|\lambda )=C^{-1}\prod_{j=1}^{p}Exp\left(o_{jj}|\frac{\lambda }{2}\right)\left\{\prod_{k=i+1}^{p}Laplace\left(o_{jk}|\lambda \right)\right\}1_{O\in M^{+}},$$

where $1_{O\in M^{+}}$ is an indicator function that **O** is positive definite, Exp(*x*|*λ*) has the exponential density of the form *p*(*x*) = *λe*^−*λx*^1_*x*>0_, and Laplace (*x*|*λ*) has the Laplace density of the form $p(x)=\frac{\lambda }{2}e^{-\lambda |x|}$. In comparison to variable selection priors such as spike-and-slab [18], the graphical LASSO prior is more scalable to high dimensions at the cost of being unable to generate estimates that are exactly zero [19]. We deal with this by using the resulting posterior samples to conclude whether a correlation is likely to be zero or not. The choice of *λ* is key to the degree of shrinkage imposed by this prior. We placed a gamma prior on *λ* in lieu of specifying it *a priori*; this is possible because [13] showed that the normalizing constant *C* does not depend on *λ*. The prior for **m** is the conditionally-conjugate normal prior N(n,L) with mean **n** and covariance matrix **L**. Hyperparameter choice for the two priors (on **m** and *λ*) is discussed in more detail below.

### Implementation and inference

BAnOCC samples the posterior using Stan’s C++ implementation and R interface [20]. Multiple quantities can be estimated from BAnOCC, including the log-basis precision, covariance, and correlation matrices. In our simulations and application, we estimated the log-basis correlation **R**_log**X**_ because it is interpretable and nicely scaled; we used the posterior median as the point estimate and the 95% credible intervals for *w*_*jk*_ to determine whether the correlation between features *j* and *k* was non-zero.

## Choosing hyperparameters

The interpretation of the prior parameters on **m** is relatively straightforward, while that of the shrinkage parameter *λ* is less clear. Because log-basis means **m** have a normal distribution, *e*^**m**^ represents the median unobserved counts, which conveniently have a log-normal distribution with parameters **n** and **L**. Therefore, we could parametrize the prior on **m** by the expected median unobserved counts **n**_*LN*_ = exp{**n** + 0.5*diag*(**L**)} and uncertainty of the median unobserved counts $L_{LN}=n_{LN}n_{LN}^{T}(e^{L}-1)$. The prior on the shrinkage parameter *λ* has a shape parameter *a* that determines how much prior probability mass is placed on *λ* values close to zero, and a rate parameter *b* that determines how the probability mass is spread across the entire domain. In particular, *a* ≤ 1 forces an asymptote at zero, while *a* > 1 does not.

When little or no prior data is available, weakly informative priors can be used. Any prior on *λ* should have high probability mass close to zero and so should have *a* ≤ 1. Larger values of *a* will “soften” the asymptotic behavior at zero (**S1 Fig**). The value of the rate parameter *b* should be chosen to so that most prior probability mass is on sensible values for *λ*. The degree of shrinkage implied by *λ* does not appreciably change for *λ* > 1 (**S2 Fig**), and so a *b* of around 5 will give a reasonable uninformative prior distribution for *λ*. For the log-basis means, m∼N(0,lI) can be used, with *l* a large value such as 100. An overlarge value for *l* can make computation less efficient and put prior mass on grossly implausible values of e^**m**^, so an *l* of 500 or less is reasonable.

Prior subject-matter information can be incorporated into the priors for both *λ* and **m**, but most easily into the prior on **m**. If the data have few features, a smaller shape hyperparameter *a* should be employed to upweight values of *λ* that yield high shrinkage. The implied prior on the median unobserved counts *e*^**m**^ could be sampled to provide an empirical distribution of the total counts $\overset{p}{\underset{j=1}{\sum }}e^{m_{j}}$; this could be assessed for gross deviations from what might be considered reasonable, or agreement with known ranges if such data are available.

## Software

The implementation of BAnOCC is publicly available with source code, documentation, and tutorial data as an R/Bioconductor package at http://huttenhower.sph.harvard.edu/banocc.

## Results

### Unobserved count mean and covariance determine spurious correlation sign and magnitude

We first aimed to identify what characteristics of compositional data impede or facilitate the accurate estimation of the unobserved count correlation matrices in general. Such characteristics should delineate when BAnOCC or any other technique for estimating the unobserved count correlation would perform well. A first-order Taylor expansion approximates the compositional covariance as a function of the mean and covariance of the unobserved counts. Because the compositional correlation is a function of the compositional covariance, the resulting approximation also explains how the correlation behaves. Letting **X** represent the unobserved counts and **C** the composition, with the mean of **X** denoted by **μ**_*X*_ = (*μ*_*X*,*j*_)^*T*^ and the approximate average proportions by $\omega ={\left(\frac{\mu_{X,1}}{\overset{p}{\underset{j=1}{\sum }}\mu_{X,j}},\ldots ,\frac{\mu_{X,p}}{\overset{p}{\underset{j=1}{\sum }}\mu_{X,j}}\right)}^{T}$, the Taylor expansion yields

$$\Sigma_{C}\approx {\left(\frac{1}{\overset{p}{\underset{j=1}{\sum }}\mu_{X,j}}\right)}^{2}(I-\omega 1^{T})\Sigma_{X}(I-\omega 1^{T}{)}^{T}.$$ (2)

Here **I** is the *p* × *p* identity matrix, and **1** is a *p*-dimensional vector of 1’s. Eq (2) allows us to approximate the behavior of the compositional covariance from the parameters of the unobserved counts that generate it. For a detailed derivation, see **S1 Text**.

### Spurious correlation can take any value between -1 and 1

**Fig 2:**
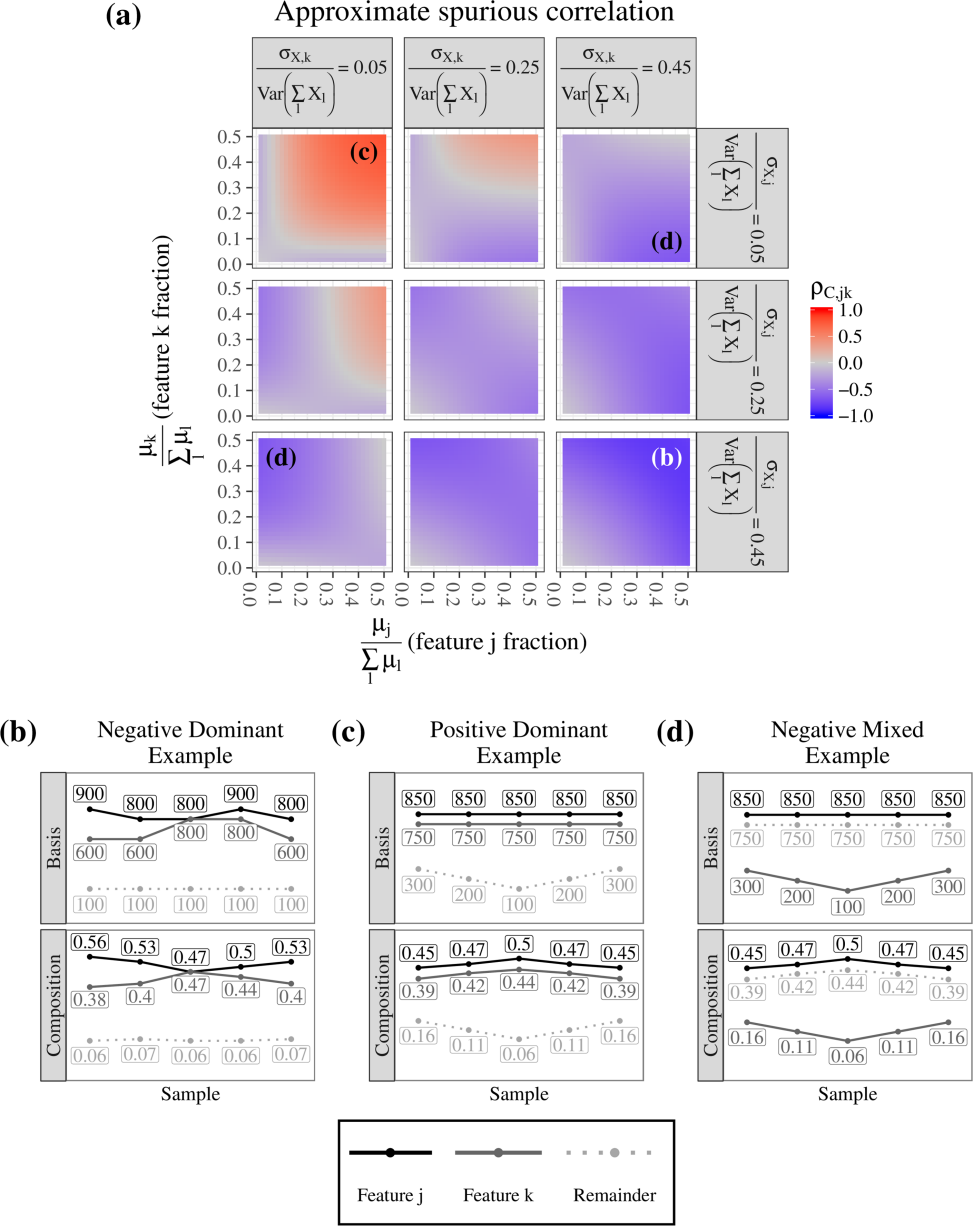
Spurious correlation is not constrained as a function of feature count, mean, and variance. **A** The approximate compositional correlation (based on Eq (3)) between features j and k when σ_X,jk_ = 0, as a function of the proportion of the total mean and proportion of total variability they contribute. **B**-**D** Examples of compositions that display positive (**B**) and negative (**C**-**D**) compositional correlations; in each, the top panel shows the correlation of the unconstrained and unobserved abundances across samples, while the bottom panel shows the correlation of the relative abundances across samples. The spurious correlation can be positive or negative, and of arbitrary magnitude, depending on the characteristics of the unobserved abundances.

Surprisingly, when no features are correlated on the unobserved count scale, the spurious correlation can take any value in [−1,1] depending on the properties of the unobserved counts (**Fig 2**). This is suggested by considering Eq (2) when *σ*_*X*,*jk*_ = 0 for all *j* ≠ *k*, then

$$\sigma_{C,jk}\approx {\left(\frac{1}{\overset{p}{\underset{l=1}{\sum }}\mu_{X,l}}\right)}^{2}\left[\omega_{j}\omega_{k}\underset{l}{\sum }\sigma_{X,ll}-\omega_{j}\sigma_{X,kk}-\omega_{k}\sigma_{X,jj}\right]\;for\;j\neq k.$$ (3)

The weights *ω*_*j*_ and the variances *σ*_*X*,*ll*_ can be configured arbitrarily to force *σ*_*C*,*jk*_ either to the extreme positive or extreme negative end of the spectrum. In particular, we see three types of strong spurious correlations (**Fig 2B–2D**): “negative dominant”, “positive dominant”, and “negative mixed”. These three types of correlations are thus representative of a range of expected real-world behaviors, and we included them in subsequent simulation studies of BAnOCC and previous models.

“Negative dominant” spurious correlation (**Fig 2B**) occurs when features *j* and *k* in the unobserved counts have (1) high mean and (2) high variability compared to the remaining (*l* ≠ *j*,*k*) features. Intuitively, the remaining features must contribute minimally to the total mean or total variance in the unobserved counts. When normalized, the sum-constraint thus forces a negative correlation between features *j* and *k* because they behave as if they were the only two features in the composition.

In the “positive dominant” spurious correlation type (**Fig 2C**), features *j* and *k* in the unobserved counts have (1) small variability and (2) high mean relative to the remaining (*l* ≠ *j*,*k*) features. The positive correlation in the composition results because the variability in the sum of the remaining feature abundances causes the compositions for features *j* and *k* to be shrunk or stretched in the same direction when the data are normalized.

Finally, “negative mixed” spurious correlations are the result of “positive dominant” type bases where feature *k* and the remaining features have switched roles (**Fig 2D**). After normalization, the variability in feature *k* forces feature *j* to move in the opposite direction to accommodate the remaining features.

### Extending and improving current assumptions about compositional correlation

Eq (3) also offers an alternative explanation for the negative covariance between features in a Dirichlet distribution. A Dirichlet distribution with parameters *α*_1_…,*α*_*p*_ results when each feature is independent on the unobserved count scale and has a *Gamma*(*α*_*j*_,*β*). The mean and variance of a Gamma distribution are $\frac{\alpha }{\beta }$ and $\frac{\alpha }{\beta^{2}}$, respectively, implying that in the unobserved counts, a feature with high mean will also have high variance, and vice versa. This captures “negative dominant” correlations well, but fails to capture “positive dominant” or “negative mixed” correlations, which result when at least one feature has high mean but *low* variance in the unobserved counts.

Eqs (2) and (3) further suggest that the overall effect of normalization on the correlation estimate as the number of features *p* increases depends on the characteristics of **μ**_*X*_ and **Σ**_*X*_. In ecological applications, it is often assumed that if *p* is large and the compositional means are similar across the *p* features, then the correlation estimates based on the composition and unobserved counts are not likely to be very different [8,10]. Part of the appeal of this reasoning is that it does not rely on information about the unobserved and unconstrained counts. Expanding Eq (2), we can see that **Σ**_*C*_ ∝ **Σ**_*X*_ − **ω1**^*T*^**Σ**_*X*_ − **Σ**_*X*_**1ω**^*T*^ + **ω1**^*T*^**Σ**_*X*_**1ω**^*T*^. If the means are very similar to each other, this affects only the weights **ω** given to the offset **ω1**^*T*^**Σ**_*X*_ − **Σ**_*X*_**1ω**^*T*^ + **ω1**^*T*^**Σ**_*X*_**1ω**^*T*^. Small weights render the offset negligible only in the case where the unobserved variance on the unobserved counts **Σ**_*X*_ is not too large: the behavior of the offset as the number of features increases depends on the similarity of the means (through **ω**) *and* on the variances of the additional features in the unobserved counts (through **Σ**_*X*_).

Thus when analyzing compositional data, one cannot know with certainty in which data the correlations are strongly affected by the normalization, much less the magnitude and direction of the change in correlation structure induced by normalizing. The information loss due to normalization implies that **Σ**_*X*_ is non-identifiable without assumptions about its structure. However, knowing how the unobserved and unobserved counts affect the spurious correlation allows simulation of datasets that have specific types of spurious correlation for testing the performance of estimation methods in these cases.

## Simulation studies

### Data generation methods

Using the information from this theoretical analysis, we tested BAnOCC on two types of datasets. The first comprised small datasets generated using the model itself but designed to be challenging by incorporating negative dominant correlations. Second, we also simulated larger, more realistic datasets using an independent model specific to microbial community structure, sparseDOSSA [21].

For the former, four small datasets with 1,000 samples and nine features each were generated according to four scenarios. The “simple” scenario had no true correlations and no negative dominant correlation; the “high spurious” scenario had no true correlations but the presence of a negative dominant correlation; the “retained spike” scenario had several true correlations and no negative dominant correlation; and the “reversed spike” scenario had several true correlations and a negative dominant correlation between two features that are positively correlated in the unobserved abundances (see details in **S2 Text** and data in **S1 Data**). On these data, we used hyperparameters *n*_*j*_ = 0, **L** = 1000**I**, *a* = 0.5 and *b* = 5 (**S3 Fig**).

Realistic data were generated using the SparseDOSSA model [21], which generates each feature from a zero-inflated, truncated log-normal distribution with subsequent rounding and estimates the feature-specific parameters by fitting to a given real-world template dataset. We induced correlations between features by using a multivariate distribution with a log-basis correlation that had off-diagonal elements set to one of four different correlation strengths ({−0.7,−0.3,0.3,0.7}). To ensure that strong compositional effects were present, we used a template with low-diversity community structure [22] with 14 pseudomicrobial features. The correlations were set so that the non-zero elements of the log-basis precision matrix and the log-basis covariance matrix would be the same; we used seven correlations (see details in **S2 Text** and data in **S2 Data**). We used hyperparameters *a* = 0.5, *b* = 5, *n*_*j*_ = 3, and **L** = 30**I** (**S4 Fig**).

### BAnOCC and CCLasso perform comparably in difficult scenarios

**Fig 3:**
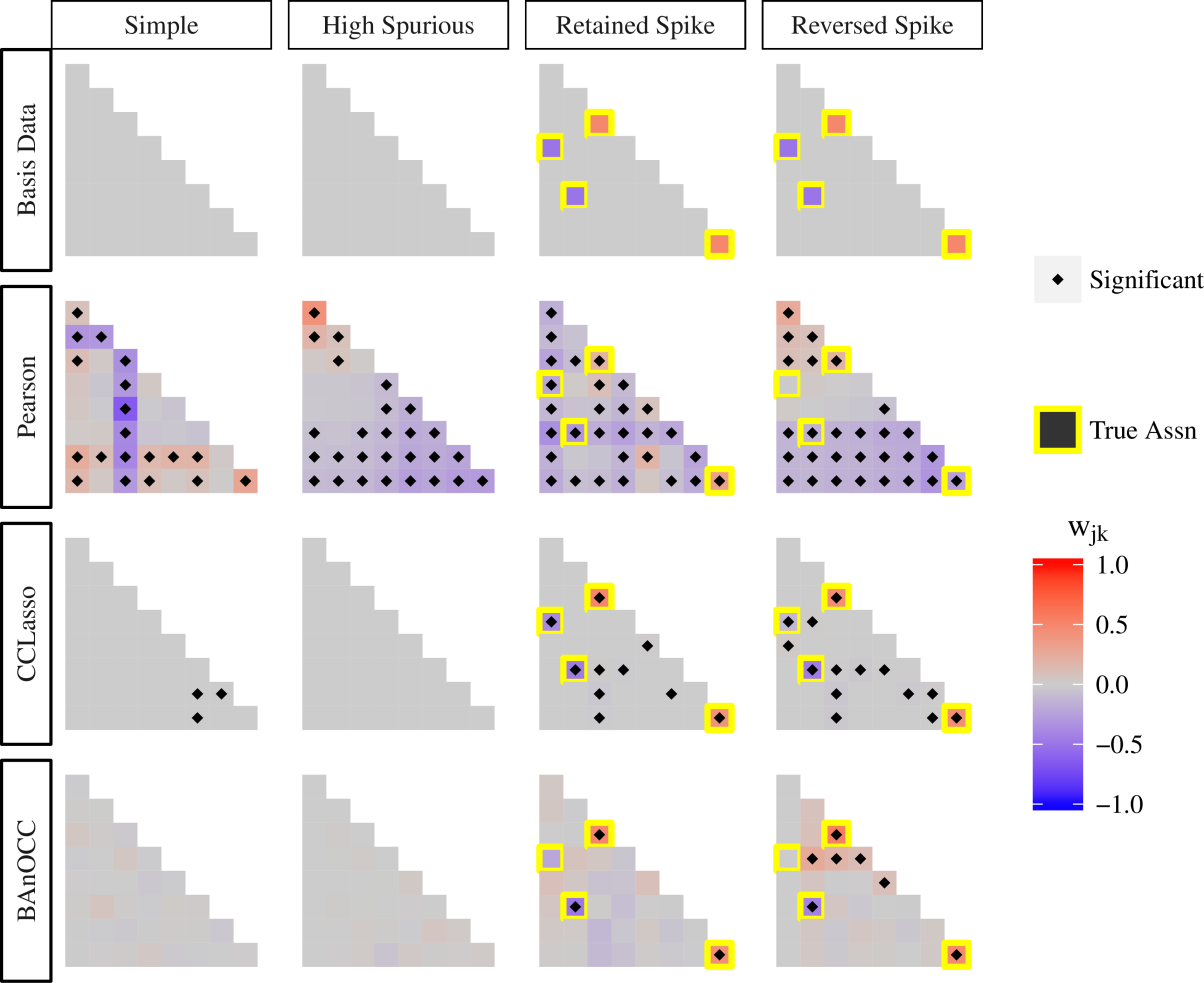
BAnOCC infers the correct unobserved abundance correlation matrix in four scenarios simulated to be challenging. Each column represents one four datasets simulated to evaluate methods for identification of correlations from compositional data: “simple”, with no true correlations and no negative dominant correlation; “high spurious”, with no true correlations and the presence of a negative dominant correlation; “retained spike” with several true correlations and no negative dominant correlation; and “reversed spike” with several true correlations and a negative dominant correlation between two positively correlated features. The top row shows the true correlation matrix. The second row shows the uncorrected compositional correlations $\overset{ˆ}{R_{C}}$ as estimated using the 1,000 samples in the simulated data. Each of the subsequent rows shows the log-basis correlation estimate ${\hat{R}}_{logX}$ and the associated inference using the compositional data for Pearson correlation, BAnOCC, and CCLasso, respectively.

Using our first set of simulated data for evaluation, we compared the estimation and inference from BAnOCC with that from CCLasso [9], a frequentist LASSO-based method that chooses the shrinkage parameter using *K*-fold cross validation (**Fig 3**). BAnOCC had much lower false positive rates than CCLasso, resulting from the model’s ability to use the posterior distribution to account for estimate uncertainty while CCLasso, being LASSO-based, used a non-zero point estimate to determine significance of an effect.

BAnOCC and CCLasso both estimate the log-basis correlation matrix accurately, and both are a substantial improvement on a naïve approach (row 2 of **Fig 3**). In particular, both BAnOCC and CCLasso have much lower false positive rates than Pearson correlation. Over all the null associations, Pearson correlation had a staggering false positive rate of 82%; CCLasso had almost 14% false positives as a result of many small but non-zero estimates; BAnOCC, because it uses the posterior credible intervals to evaluate uncertainty, had a false positive rate of about 3%. BAnOCC cannot estimate the log-basis correlations *w*_*jk*_ to be exactly zero because of the continuous prior, but the null associations whose 95% credible intervals cover zero have very small estimates (all are less than 0.15, 75% are less than 0.05).

The association between features 1 and 5 in the “reversed spike” dataset was difficult for both BAnOCC and CCLasso. Both gave a small, negative estimate (-0.001 for BAnOCC and -0.113 for CCLasso). BAnOCC displays a slight bias toward positive correlations instead of the moderate negative correlation that was present in the underlying unobserved abundances, as shown by several false positive associations in this dataset. This behavior is common among many methods, including SparCC and SPIEC-EASI (**S5 Fig**). It results from the fact that when a negative-dominant structure is present, positive correlations become much more likely to be real than negative ones, an interesting observation to consider when interpreting real-world results from any of these methods.

BAnOCC and CCLasso agree well with the true magnitude and direction of the non-zero associations that both methods conclude are significant. For these associations, the relative difference with the true value is less than 15% for both methods. When the associations were rejected, the 95% credible interval from BAnOCC covered the true value, indicating its utility for evaluating the uncertainty of the estimate. The false negative rates were 25% for BAnOCC and 0% for CCLasso, a direct result of the higher tolerance for false positives CCLasso exhibits. In practice, this has the expected effect of dramatically lowering BAnOCC’s false positive rate in recovering true correlations from compositional data.

### Comparison of type I and type II error rates

**Fig 4:**
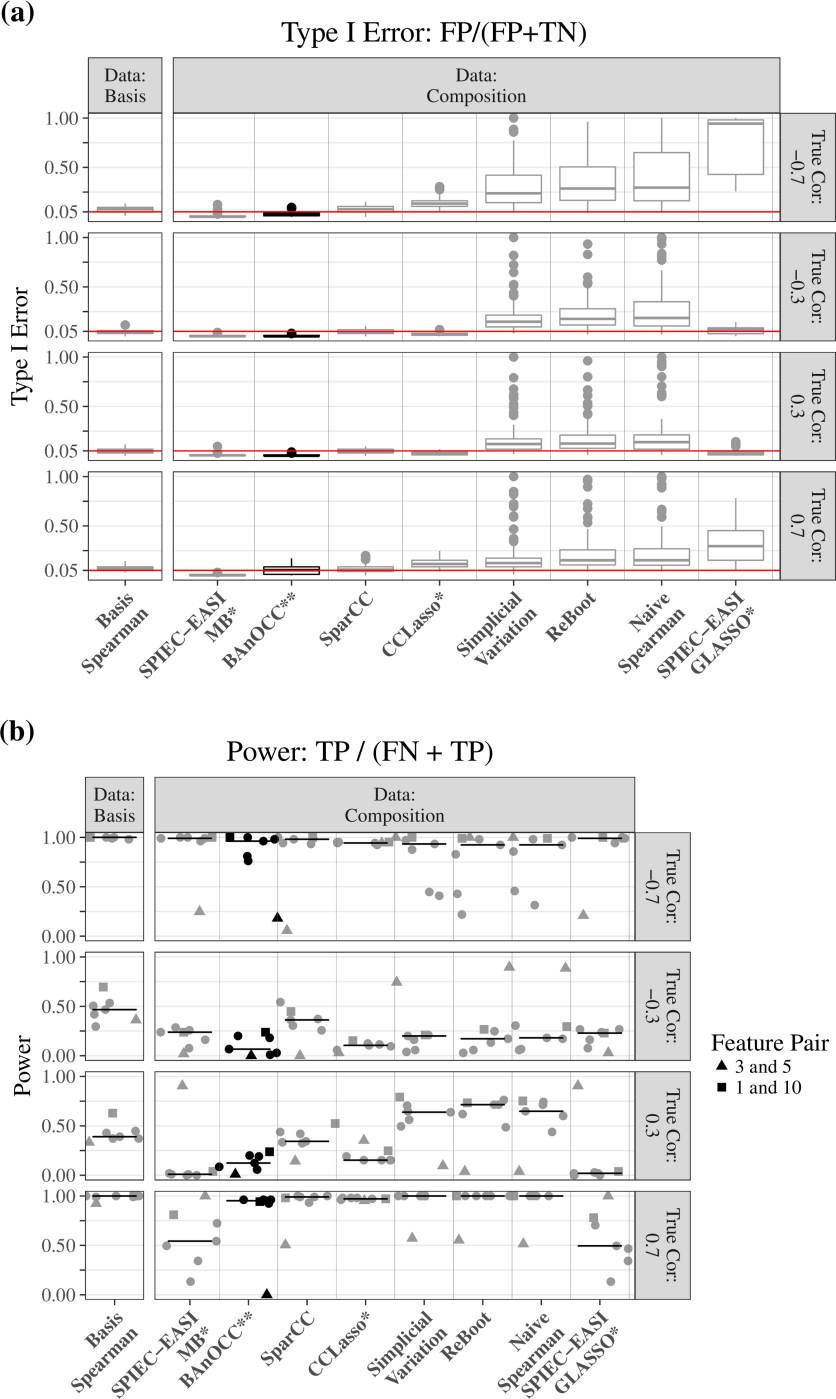
The BAnOCC model controls type I error while maintaining power. Results on simulated data comprising SparseDOSSA-derived compositions modeled on a low-diversity dataset with 14 features. The type I error rate is controlled at the 0.05 level for BAnOCC and approximately so for SparCC, CCLasso, and SPIEC-EASI (MB), but not for simplicial variation or Spearman correlation (on the composition, a negative control). BAnOCC maintains good power across all true correlation values, but as expected has better power for stronger true correlation values. Type I and type II error rates are determined by correct or incorrect rejection of H_0_ based on inference (simplicial variation, SparCC, Spearman correlation, and BAnOCC) or estimation (CCLasso and SPIEC-EASI). * = rejection of H_0_ based on estimation; ** = rejection of H_0_ based on inference from credible intervals; all others, rejection of H_0_ based on inference from p-values. (**S6 Fig** and **S7 Fig**).

We compared BAnOCC’s performance as measured by type I and type II error rates to a range of previous methods (**Fig 4**): simplicial variation [23], SparCC [8], CCLasso [9], SPIEC-EASI [11], ReBoot [7], and Spearman correlation (directly on the composition as a negative control). Of the two frequentist LASSO-based methods (CCLasso and REBACCA [10]), CCLasso alone had an R package interface; because they employ highly similar approaches, they should yield similar results. For a positive control, we also applied Spearman correlation to the unconstrained (and usually unobserved) counts (**Table 1** and **S3 Text**).

**Table 1:** Methods included in an evaluation on simulated data. Type I and type II error rates were determined for these methods by the correct or incorrect rejection of H_0_; for CCLasso and SPIEC-EASI, no inferential methodology was provided and so the correct or incorrect estimation of w_jk_ as zero was used. Note that although SPIEC-EASI infers the precision matrix, construction of the true correlation matrix in the simulated data guarantees that the same elements will be non-zero in the precision and covariance matrix.

Method	H_0_	Error calls	Inference Method
Simplicial Variation	$\frac{1}{t_{jk}}=0$	inference	one-sided permutation test
SparCC	*w*_*jk*_ = 0	inference	authors’ bootstrap-based method
CCLasso	*w*_*jk*_ = 0	estimation	-
SPIEC-EASI	*w*_*jk*_ = 0	estimation	-
ReBoot	*ρ*_*X*,*jk*_ = 0	inference	permutation-based test
BAnOCC	*w*_*jk*_ = 0	inference	95% credible interval
Spearman (composition)	*ρ*_*X*,*jk*_ = 0	inference	two-sided permutation test
Spearman (unconstrained counts)	*ρ*_*X*,*jk*_ = 0	inference	two-sided permutation test

Overall, BAnOCC controlled the type I error rate for all correlation strengths (**Fig 4A**) while maintaining comparable power compared with other recent methods (**Fig 4B**). These results held true in a more even community with larger features, in which BAnOCC was the sole method to fully control the type I error rate (**S8 Fig**). As the number of samples increased, all methods increased in power (**S9 Fig**), while the type I error rates remained fairly constant (**S10 Fig**).

Only BAnOCC and SparCC controlled type I error while maintaining high power for all correlation strengths (see also AUC boxplots in **S6 Fig**). Both behaved similarly to Spearman correlation applied to the unconstrained abundances, which represents the best possible performance (as it uses the unconstrained data rather than the composition—this is impossible in practice, when only the composition is available). SparCC’s type I error rate was slightly inflated in a larger dataset with more features, while BAnOCC continued to control the type I error rate at the nominal level (**S8 Fig**). As other authors have noted, SparCC does not guarantee that its log-basis correlation estimate has bounded elements nor that it is positive definite [9]. By contrast, BAnOCC not only estimates a positive definite correlation matrix with bounded elements, but also can infer network edges based on the precision matrix as well.

Several methods proved to control the type I error rate poorly: Spearman correlation exemplifies this as a negative control, but simplicial variation, SPIEC-EASI using GLASSO and to a lesser extent CCLasso were comparable. ReBoot, by design, attenuates the type I error rate of Spearman correlation, but does not control it perfectly. The high type I error rates are also somewhat expected in simplicial variation, but SPIEC-EASI using GLASSO may not be performing as expected, especially since in contrast the Meinshausen-Bühlmann neighborhood selection method did control type I error. This may also possibly be because the neighborhood selection infers each element of the matrix one at a time, while GLASSO infers the matrix all at once; this makes the GLASSO optimization a more difficult problem.

Feature 5 in the template dataset has a large mean and variance, while feature 3 has a small mean and variance. This results in a strong negative spurious correlation in the composition, which gives rise to interesting behavior of essentially all methods when detecting this association. When the true association is negative, many compositionally-appropriate methods such as BAnOCC, SparCC, and SPIEC-EASI (MB) do poorly at detecting the true correlation (**Fig 4B**) because the negative correlation is difficult to attribute to the unobserved counts rather than spurious correlation. Conversely, more naïve methods such as simplicial variation and Spearman correlation do very well at detecting a weak negative correlation between these two features because this becomes a strong negative correlation in the composition. This simulated example thus provides some insight into the form of sensitivity / specificity tradeoff that applies in the constrained, information-loss setting of identifying true correlations from compositions.

## A microbial interaction network from the Human Microbiome Project

**Fig 5:**
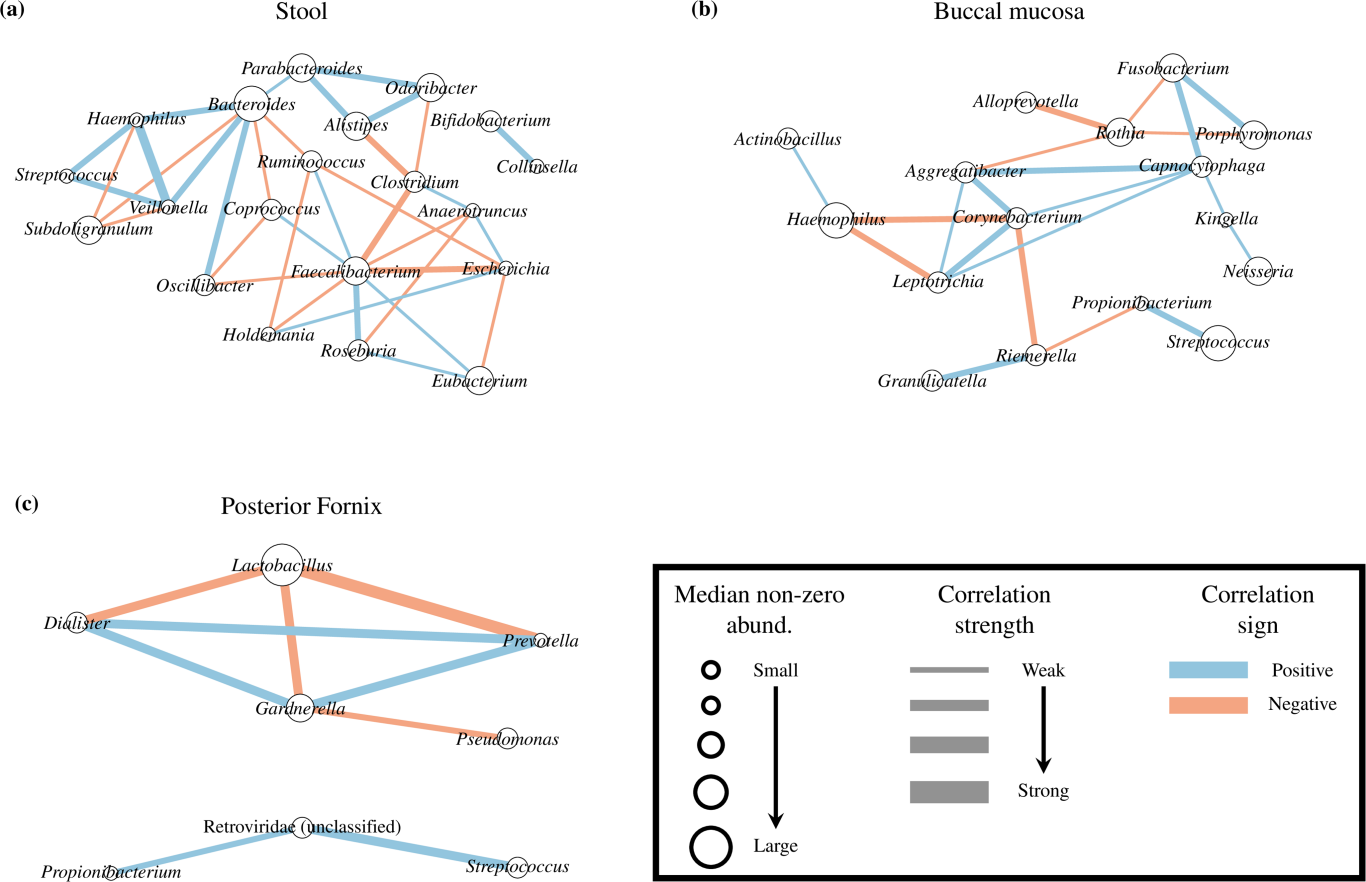
BAnOCC association networks from the Human Microbiome Project. The association networks inferred from three HMP body sites: stool (**A**), buccal mucosa (**B**), and posterior fornix (**C**). Using four chains with a minimum of 5000 iterations, we ran BAnOCC until convergence (see details in **S3 Text**). Only significant correlations stronger than 0.15 are shown (see **S1 Table**, **S2 Table** and **S3 Table**). The GLASSO prior results in sparse networks for these datasets, highlighting individual associations between taxa.

As an example application, we inferred a correlation network among microbial taxa profiled using ecological data from the Human Microbiome Project [22] (**Fig 5**). Microbial community sequencing generates compositions by assigning sequencing reads to microorganisms; since nucleotide sequencing depth is arbitrary, the resulting counts are not informative regarding the unobserved and unconstrained counts and are often normalized to relative abundances. Co-variation patterns in such data are of interest because they suggest ecological interactions, such as mutualism (positive correlation) or predation (negative correlation) [7].

The microbial taxonomic relative abundance data used here consisted of 523 microbial features measured across 700 total samples using MetaPhlAn2 v2.0_beta1 [24] in July of 2014 (available in **S3 Data**), further excluding from all networks markers removed in the subsequent version’s database (v2.0_beta2). These samples were in turn drawn from 127 individuals at six distinct body sites. Microbial ecology differs at each body site [22], providing examples for BAnOCC analysis that ranged from diverse, relatively even communities (such as stool) to less diverse, highly skewed ecologies (such as the vaginal posterior fornix). For each of three representative body sites (stool, posterior fornix, and buccal mucosa), we selected the first time point from each subject, collapsed taxonomic information to the genus level, and then removed features with relative abundance less than 0.0001 in at least 50% of samples. With too few features, little to nothing can be concluded about the true correlations; so if fewer than 10 features remained we lowered the prevalence cutoff until 10 features were retained.

The hyperparameters for the gamma prior on *λ* were *a* = 0.5 and *b* = 5 for all body sites, ensuring that we gave substantial weight to sparser precision matrices. For all body sites, we used the prior variability of the log-basis means **L** = 30**I**; each body site, however, had a different *n*_*j*_ so that the distribution of the sums of medians were similar across different body sites (see **S11–S14 Figs**). We further compared BAnOCC’s inferred network using the log-basis correlation matrix with that from CCLasso, and BAnOCC’s inferred network using the log-basis precision matrix with SPIEC-EASI. There is broad agreement between the methods as to which edges are significant, with very few edges discrepant between the methods (**S15 Fig**).

In stool, BAnOCC inferred several positive associations between genera within the family Bacteroidales, in particular *Bacteroides*, *Odoribacter*, *Parabacteroides* and *Alistipes* (**Fig 5A**). Until recently, these genera were classified as part of the same genus [25]. This supports the common observation that closely (but not too closely) related taxa tend to have positive ecological associations [26]. Additionally, positive associations in the buccal mucosa (**Fig 5B**) connect taxa that are known to physically co-aggregate; in particular, *Fusobacterium* interactions with species from the *Porphyromonas* and *Capnocytophaga* genera (among others) are crucial in biofilm formation [27] and have been previously recovered from 16S-based ecological analyses [7]. Lastly, we can see the well-documented negative association between the *Lactobacillus* genus in the posterior fornix with several genera associated with dysbiosis such as *Gardnerella* and *Prevotella* [28] (**Fig 5C**).

Two interactions newly suggested by this analysis involved the Proteobacteria across multiple body sites, and specifically in stool and the oral cavity (buccal mucosa). The genera *Escherichia* and *Haemophilus* represent the two major proteobacterial residents in these habitats, respectively, and both were involved in predominantly negative interactions with more typical, abundant members of these communities (e.g. *Faecalibacterium* and *Eubacterium* in the gut, *Leptotrichia* or *Corynebacterium* in the mouth). These clades are highly phylogenetically diverged and tend to carry larger, more generalize genomes and pan-genomes [29,30]; this suggests that they will overgrow in these habitats only in unusual situations, exemplified by *E*. *coli*’s abundance in the gut primarily during inflammation [31]. Further details may be provided by future analyses using BAnOCC or related methods on species or strain-level ecological profiling.

## Discussion

Here, we describe BAnOCC, a Bayesian method for inferring the log-basis correlation structure from compositional data. Assuming a log-normal distribution on the unobserved and unconstrained counts, the model estimates the log-basis correlations using a sparsity-inducing shrinkage prior on the log-basis precision matrix. It is part of a family of several recently proposed LASSO-based methods [9–11] which provide a more rigorous approach to correcting for compositional effects than earlier methods [7,8]. Unlike the other LASSO-based correlation-inference methods that summarize pairwise associations using a single point estimate, BAnOCC yields uncertainty estimates of the precision, covariance, and correlation parameters. Simulation results show that BAnOCC performs as well as or better than existing methods in controlling type I error while maintaining power for network edge detection from compositional data. Finally, we applied the method to assess microbial relationships in the human microbiome, confirming established interactions and suggesting novel ones for future validation.

Analysis using a Taylor series approximation provided one of the first characterizations of properties that make true correlations “difficult” to recover from compositions, or conversely “easy” to miss as false negatives. In particular, this depends not only on the more intuitive number and evenness of feature means, but also on the distribution of their variance. This allowed us to simulate designedly difficult test cases for BAnOCC and a variety of published methods, in contrast to previous simulation studies that relied primarily on relatively simple synthetic data [7–10]. In most studies, spurious correlation is noted to be commonly present and of varying magnitudes and directions [11]. However, the possible sensitivity of methods to the type of spurious correlation encountered has not been explored and is an important contribution to the characterization of existing and future methods.

We anticipate several computational and statistical refinements that may further improve BAnOCC’s performance. While BAnOCC uses 95% credible intervals for inference, these can be overly conservative [32]. Alternative thresholding methods may improve on this, such as the scaled neighborhood criterion [32] or the partial-correlation based approach of [33] and [13]. A discrete-continuous mixture prior such as the *G* -Wishart prior [34] or the covariance selection prior [35] on the log-basis correlation matrix would further allow the posterior probability that *w*_*jk*_ = 0 to be nonzero, and this quantity could be used as a threshold.

For applications specifically on count data, such as microbial compositions, the data could be modeled more accurately by adding a hierarchical layer. This would generate measurement counts conditional on the unobserved and unconstrained counts, making the observed compositions a function of normalized measurement counts. The degree of zero-inflation observed in ecological data could also be modeled directly using a hurdle or mixture model, or a multinomial distribution for the measurement counts. This would provide a particularly targeted approach for microbial ecology, in which more detailed data (at the species or strain level [24]) could be further incorporated. We thus hope to refine both the accuracy of compositional correlation inference and the applications to microbial community data in future studies.

## Supporting information

S1 Text Detailed mathematical derivations. Beginning from initial definitions, a step-by-step derivation of the likelihood in Eq (1), the marginal likelihood for the composition, and the Taylor Series approximation in Eq (2).
(DOCX)

S2 Text Detailed description of simulated datasets. Descriptions of how the datasets were generated for both the challenging scenarios case and for the realistic data case.
(DOCX)

S3 Text Implementation of methods compared. Details on how each of the methods compared in the **Results** section were implemented, run on the simulated data, and evaluated for type I and type II errors.
(DOCX)

S1 Data Simulated data for difficult scenarios. The simulated data for each of four difficult simulation scenarios described in the **Results** section. For details on how these were generated, see **S2 Text**.
(ZIP)

S2 Data Realistic simulated data. All simulated datasets from sparseDOSSA, as well as the template dataset used. For details on how these were generated, see **S2 Text**.
(ZIP)

S3 Data HMP taxonomic profiles. The taxonomic profiles from the Human Microbiome Project data as processed with MetaPhlAn version 2.0_beta1 [24].
(ZIP)

S1 Fig A relatively informative prior on λ is effective. The densities of different priors on λ for different ranges of λ values. The shape parameter a determines how quickly the prior density decreasys, while the rate parameter b determines how much prior weight is placed on small λ values rather than large λ values.
(TIF)

S2 Fig Shrinkage increases for smaller λ. **A** The shape of the prior on o_jk_ and o_jj_ for several values of λ. Smaller λ results in greater shrinkage towards zero. **B**-**C** The prior probability in the interval (−0.001,0.001) for each off-diagonal element o_jk_| λ∼Laplace(λ) across small (**B**) or large (**C**) values of λ. Small values (<0.1) of λ show the greatest shrinkage, while beyond λ = 1 the shrinkage becomes negligible, as shown by the maximal shrinkage for λ > 0.2 being <0.005.
(TIF)

S3 Fig Prior distributions for test cases. The prior distributions for the test cases used a prior on **m** that was very uninformative, being centered at 0 and with a large variance. The prior on λ put most prior weight on λ values less than one and had narrow tails to encourage shrinkage of the correlation estimates (**B**).
(TIF)

S4 FigPrior distributions for realistic simulated data.We used a prior for **m** that gave reasonable behavior for the sum of the unobserved count medians $\overset{14}{\underset{j=1}{\sum }}e^{m_{j}}$ (**A**). The prior on λ put most prior weight on λ values less than one and had narrow tails to encourage shrinkage of the correlation estimates (**B**). (See also **S14 Fig**).(TIF)

S5 FigAdditional results for difficult scenarios.The estimates and significance of several methods on the four scenarios (columns): simple, with no true correlations and no negative dominant spurious correlation; high spurious, with no true correlations and a negative dominant spurious correlation; retained spike, with several true correlations and no negative dominant spurious correlation; and reversed spike, with several true correlations and a negative dominant spurious correlation. The top row is data-derived, with the bottom triangle indicating the true log-basis correlation ${\hat{R}}_{logX}$ and the top triangle the compositional correlation calcualted using the 1,000 samples from the data. BAnOCC evaluates significance using 95 % credible intervals. CCLAsso and SPIEC-EASI (MB) are significant if they are non-zero. SPIEC-EASI (MB) colors indicate the sign rather than the magnitude of the estimated correlations as the estimates are not possible to compute. SparCC evaluates significance using a bootstrap-based method. All the methods do poorly at detecting and correctly estimating the negative correlation between features 1 and 5 in the reversed spike scenario, and instead tend to falsely detect several positive correlations.(TIF)

S6 Fig AUC boxplots of method performance on “realistic” simulated datasets. For each given correlation strength and template dataset, AUCs were calculated for each of 105 simulated datasets comprising sparseDOSSA-derived compositions with 100 samples modeled on a low-diversity dataset with 14 features. The ROCs used to measure the AUCs are based on p-values (Spearman correlation, simplicial variation, SparCC), credible intervals (BAnOCC), correlation estimate (CCLasso) or stability score (SPIEC-EASI). Thus each boxplot consists of 105 points. Each of the 105 AUCs are measured over seven true correlations, and all of the methods do better than expected by chance (red line), although BAnOCC has overall the highest average AUC.
(TIF)

S7 Fig Average ROC curves of method performance on “realistic” simulated datasets. For a given correlation strength, each ROC is calculated over the aggregation of all 735 true associations in 105 simulated datasets comprising SparseDOSSA-derived compositions with 100 samples modeled on a low-diversity dataset with 14 features. The cutoffs used are based on p-values (Spearman correlation, simplicial variation, SparCC), credible interval width (BAnOCC), correlation estimate (CCLasso) or stability score (SPIEC-EASI).
(TIF)

S8 Fig Type I error rates and power in large datasets. Results on simulated data comprising 100 SparseDOSSA-derived compositions modeled on a high-diversity dataset with 89 features. **A** Type I error rates are controlled across all correlation values only by BAnOCC. **B** Power is comparable between BAnOCC and other modern methods across spiked correlation strengths, with BAnOCC and others correctly controlling error rates and only BAnOCC providing full inference and probability distributions on the resulting microbial interaction networks. * = rejection of H_0_ based on estimation; ** = rejection of H_0_ based on inference from credible intervals; all others, rejection of H_0_ based on inference from p-values. (See **S16 Fig** for the priors used.)
(TIF)

S9 Fig Power across multiple sample sizes and numbers of features. Power on simulated data comprising SparseDOSSA-derived compositions modeled on a low-diversity dataset with 14 features (small template) or a high-diversity dataset with 89 features (large template). See **S2 Text** for simulation details. The rows correspond to the number of samples (50, 100, or 150) simulated. BAnOCC controls the type I error rate in all scenarios, and the type I error rate behavior for most methods does not change with increasing sample size. * = rejection of H_0_ based on estimation; ** = rejection of H_0_ based on inference from credible intervals; all others, rejection of H_0_ based on inference from p-values.
(TIF)

S10 Fig Type I error rates across multiple sample sizes and numbers of features. Type I error rates on simulated data comprising SparseDOSSA-derived compositions modeled on a low-diversity dataset with 14 features (small template) or a high-diversity dataset with 89 features (large template). See **S2 Text** for simulation details. The rows correspond to the number of samples (50, 100, or 150) simulated. BAnOCC controls the type I error rate in all scenarios, and the type I error rate behavior for most methods does not change with increasing sample size. * = rejection of H_0_ based on estimation; ** = rejection of H_0_ based on inference from credible intervals; all others, rejection of H_0_ based on inference from p-values.
(TIF)

S11 FigPrior distributions for the stool body site.We used a prior for **m** that gave reasonable behavior for the sum of the unobserved count medians $\overset{24}{\underset{j=1}{\sum }}e^{m_{j}}$ (**A**). The prior on λ put most prior weight on λ values less than one and had narrow tails to encourage shrinkage of the correlation estimates (**B**). (See also **S14 Fig**).(TIF)

S12 FigPrior distributions for the buccal mucosa body site.We used a prior for **m** that gave reasonable behavior for the sum of the unobserved count medians $\overset{21}{\underset{j=1}{\sum }}e^{m_{j}}$ (**A**). The prior on λ put most prior weight on λ values less than one and had narrow tails to encourage shrinkage of the correlation estimates (**B**). (See also **S14 Fig**).(TIF)

S13 FigPrior distributions for the posterior fornix body site.We used a prior for **m** that gave reasonable behavior for the sum of the unobserved count medians $\overset{11}{\underset{j=1}{\sum }}e^{m_{j}}$ (**A**). The prior on λ put most prior weight on λ values less than one and had narrow tails to encourage shrinkage of the correlation estimates (**B**). (See also **S14 Fig**).(TIF)

S14 FigImplied priors on median unobserved counts.The implied priors on the median unobserved counts $e^{m_{j}}$ (top panel) and the sum of the median unobserved counts $\overset{p}{\underset{j=1}{\sum }}e^{m_{j}}$ (bottom panel) for the SparseDOSSA simulated data and the body sites from the application. Each distribution is estimated using 100,000 random samples. The mean of m_j_ was selected such that the sum of the median unobserved counts approximately shared the same average.(TIF)

S15 Fig Comparison of inferred networks on HMP data. The number of edges significant in both methods, neither method, or only one method, stratified by body site and whether the methods use the log-basis precision or correlation matrix. Most edges are concordantly significant (or not) between both methods; few are significant by only one method. Further, most of the edges significant in CCLasso but not BAnOCC are small in magnitude (BAnOCC not sig, CCLasso magnitude < 0.1).
(TIF)

S16 FigPrior distributions for large datasets.For our larger datasets simulated based on a stool dataset with 89 features, we used a prior for **m** that gave reasonable behavior for the sum of the basis medians $\overset{89}{\underset{j=1}{\sum }}e^{m_{j}}$ (**A**). The prior on λ put most prior weight on λ values less than one and had narrow tails to encourage shrinkage of the correlation estimates (**B**). (See also **S14 Fig**).(TIF)

S1 Table BAnOCC stool network. The significant edges from running BAnOCC on the stool body site with 5,500 warmup iterations and 12,000 total iterations. Edges are ordered by posterior median correlation magnitude. “hpd.95.ci” indicates the highest posterior density 95% credible intervals.
(XLSX)

S2 Table BAnOCC buccal mucosa network. The significant edges from running BAnOCC on the buccal mucosa body site with 5,500 warmup iterations and 12,000 total iterations. Edges are ordered by posterior median correlation magnitude. “hpd.95.ci” indicates the highest posterior density 95% credible intervals.
(XLSX)

S3 Table BAnOCC posterior fornix network. The significant edges from running BAnOCC on the posterior fornix body site with 1,500 warmup iterations and 5,000 total iterations. Edges are ordered by posterior median correlation magnitude. “hpd.95.ci” indicates the highest posterior density 95% credible intervals.
(XLSX)
