# Supplementary material for: A Bayesian method for detecting pairwise associations in compositional data
Source: PLoS Comput Biol. 2017 Nov 15;13(11):e1005852. doi: 10.1371/journal.pcbi.1005852 (PMC5706738; doi:10.1371/journal.pcbi.1005852)
Supplement: S1 Text — Beginning from initial definitions, a step-by-step derivation of the likelihood in Eq (1), the marginal likelihood for the composition, and the Taylor Series approximation in Eq (2). (DOCX) [file pcbi.1005852.s001.docx]

Detailed Likelihood Derivations

# Complete Likelihood Derivation

For a single observation, let $\mathbf{x}=(x_{1},\ldots,x_{p})$ be the unobserved and unconstrained counts and $t=\sum_{j=1}^{p} x_{j}$ be the total. Under our model, $\mathbf{x}\sim\mathcal{LN}(\mathbf{m},\mathbf{O})$. The normalized composition is $\mathbf{c}=(c_{1},\ldots,c_{p}))$, where $c_{j}=\frac{x_{j}}{t}$.

The density of $\mathbf{x}$ is given by:

$$f(\mathbf{x})=\frac{exp\left\{ \frac{-1}{2}\left[ (\log(\mathbf{x})-\mathbf{m})^{T}\mathbf{O}(\log(\mathbf{x})-\mathbf{m}) \right] \right\}}{(2\pi)^{p/2}|\mathbf{O}|^{-1/2}\prod_{j=1}^{p} x_{j}}$$

Note that $\mathbf{x}$ can be alternatively represented by $\mathbf{y}=g(\mathbf{x})=(t,c_{1},\ldots,c_{p-1})$, with inverse transformation $g^{-1}(\mathbf{y})=\left( c_{1}t,\ldots c_{p-1}t,\left( 1-\underset{\overset{p-1}{j=1}}{\sum}c_{j} \right)t \right)$ and determinant of the Jacobian $t^{p-1}$. The density of $\mathbf{y}$ is then:

$$\begin{matrix} f(\mathbf{y}) & =f(g^{-1}(\mathbf{y}))|J| \\ & =\frac{exp\left\{ \frac{-1}{2}\left[ (\log(t\mathbf{c})-\mathbf{m})^{T}\mathbf{O}(\log(t\mathbf{c})-\mathbf{m}) \right] \right\}t^{p-1}}{(2\pi)^{p/2}|\mathbf{O}|^{-1/2}\prod_{j=1}^{p} tc_{j}} \\ & =\frac{exp\left\{ \frac{-1}{2}\left[ (\log(t\mathbf{c})-\mathbf{m})^{T}\mathbf{O}(\log(t\mathbf{c})-\mathbf{m}) \right] \right\}}{(2\pi)^{p/2}|\mathbf{O}|^{-1/2}t\prod_{j=1}^{p} c_{j}} \end{matrix}$$

# Marginal Likelihood Derivation

Taking just the exponentiated portion of the numerator of the complete likelihood and letting $\mathbf{1}$ be a $p$-dimensional vector of 1’s, we see that:

$$\begin{matrix} & -\frac{1}{2}\left[ (\log(t\mathbf{c})-\mathbf{m})^{T}\mathbf{O}(\log(t\mathbf{c})-\mathbf{m}) \right] \\ & =-\frac{1}{2}\left[ (\mathbf{1}\log t+\log\mathbf{c}-\mathbf{m})^{T}\mathbf{O}(\mathbf{1}\log t+\log\mathbf{c}-\mathbf{m}) \right] \\ & =-\frac{1}{2}\left[ (\log t)^{2}\mathbf{1}^{T}\mathbf{O1}-2(\log t)(\mathbf{m}-\log\mathbf{c})^{T}\mathbf{O1}+(\log\mathbf{c}-\mathbf{m})^{T}\mathbf{O}(\log\mathbf{c}-\mathbf{m}) \right] \\ & =-\frac{1}{2}\left[ \left( \log t-(\mathbf{m}-\log\mathbf{c})^{T}\mathbf{O1}(\mathbf{1}^{T}\mathbf{O1})^{-1} \right)^{2}\mathbf{1}^{T}\mathbf{O1} \right. \\ & \left. -\left( (\mathbf{m}-\log\mathbf{c})^{T}\mathbf{O1}(\mathbf{1}^{T}\mathbf{O1})^{-1} \right)^{2}\mathbf{1}^{T}\mathbf{O1}+(\log\mathbf{c}-\mathbf{m})^{T}\mathbf{O}(\log\mathbf{c}-\mathbf{m}) \right] \end{matrix}$$

Let $\mu^{*}=(\mathbf{m}-\log\mathbf{c})^{T}\mathbf{O1}(\mathbf{1}^{T}\mathbf{O1})^{-1}$ and ${\sigma^{2}}^{*}=\frac{1}{\mathbf{1}^{T}\mathbf{O1}}$. Then the marginal likelihood becomes:

$$\begin{matrix} \int_{0}^{\infty} f(\mathbf{c},t)dt & =\int_{0}^{\infty} \frac{\exp\left\{ \frac{-1}{2}\left[ (\log(t\mathbf{c})-\mathbf{m})^{T}\mathbf{O}(\log(t\mathbf{c})-\mathbf{m}) \right] \right\}}{(2\pi)^{p/2}|\mathbf{O}|^{-1/2}t\prod_{j=1}^{p} c_{j}}dt \\ & =\int_{0}^{\infty} \frac{\sqrt{2\pi{\sigma^{2}}^{*}}\exp\left\{ -\frac{1}{2}\left[ (\log t-\mu^{*})^{2}/{\sigma^{2}}^{*}-\frac{{\mu^{*}}^{2}}{{\sigma^{2}}^{*}}+(\log\mathbf{c}-\mathbf{m})^{T}\mathbf{O}(\log\mathbf{c}-\mathbf{m}) \right] \right\}}{(2\pi)^{p/2}|\mathbf{O}|^{-1/2}\sqrt{2\pi{\sigma^{2}}^{*}}t\prod_{j=1}^{p} c_{j}}dt \\ & =\frac{\sqrt{{\sigma^{2}}^{*}2\pi}\exp\left\{ (\log\mathbf{c}-\mathbf{m})^{T}\mathbf{O}(\log\mathbf{c}-\mathbf{m})-\frac{{\mu^{*}}^{2}}{{\sigma^{2}}^{*}} \right\}}{(2\pi)^{p/2}|\mathbf{O}|^{1/2}\prod_{j=1}^{p} c_{j}}, \end{matrix}$$

because $\int_{0}^{\infty} \frac{1}{\sqrt{2\pi{\sigma^{2}}^{*}}t}\exp\left\{ -\frac{(\log t-\mu^{*})^{2}}{2{\sigma^{2}}^{*}} \right\}dt=1$.

# Taylor Series Expansion

Let $\mathbf{X}=(X_{1},\ldots,X_{p})^{T}$ represent the unconstrained counts, and for convenience, let the sum of $\mathbf{X}$ be denoted by $T=\sum_{j=1}^{p} X_{j}$. Let the composition derived from $\mathbf{X}$ be denoted by $g(\mathbf{X})=\left( \frac{X_{1}}{T},\ldots,\frac{X_{p}}{T} \right)$. Further let the mean of $\mathbf{X}$ be denoted by $\boldsymbol{\mu}_{X}=(\mu_{X,1},\ldots,\mu_{X,p})^{T}$ and the variance of $\mathbf{X}$ by $\boldsymbol{\Sigma}_{X}=[\sigma_{X,jk}]$.

A first-order Taylor series expansion allows us to approximate $g(\mathbf{X})$ as a linear function at $\boldsymbol{\mu}_{X}$:

$$g(\mathbf{X})\approx g(\boldsymbol{\mu}_{X})+\nabla g(\boldsymbol{\mu}_{X})(\mathbf{X}-\boldsymbol{\mu}_{X})$$

This implies that

$$Var(g(\mathbf{X}))\approx Var(g(\boldsymbol{\mu}_{X})+\nabla g(\boldsymbol{\mu}_{X})(\mathbf{X}-\boldsymbol{\mu}_{X}))=\nabla g(\boldsymbol{\mu}_{X})Var(\mathbf{X})\nabla g(\boldsymbol{\mu}_{X})^{T}$$

Generally, $\frac{\partial}{\partial X_{j}}g_{k}(\mathbf{X})=\frac{1}{T}I(j=k)-\frac{X_{j}}{T^{2}}$, where $I(\cdot)$ is the identity function. Thus the gradient of the transformation $g(\mathbf{X})$ is given by:

$$\begin{matrix} \nabla g(\mathbf{X}) & =\left[ \begin{matrix} \frac{\partial}{\partial X_{1}}g_{1}(\mathbf{X}) & \ldots& \frac{\partial}{\partial X_{1}}g_{p}(\mathbf{X}) \\ \vdots& \ddots& \vdots\\ \frac{\partial}{\partial X_{p}}g_{1}(\mathbf{X}) & \ldots& \frac{\partial}{\partial X_{p}}g_{p}(\mathbf{X}) \end{matrix} \right] \\ & =\left[ \begin{matrix} \frac{1}{T}\left( 1-\frac{X_{1}}{T} \right) & \ldots& \frac{-X_{1}}{T^{2}} \\ \vdots& \ddots& \vdots\\ \frac{-X_{p}}{T^{2}} & \ldots& \frac{1}{T}\left( 1-\frac{X_{p}}{T} \right) \end{matrix} \right] \\ & =\frac{1}{T}\left[ \mathbf{I}-\frac{1}{T}\mathbf{X}\mathbf{1}^{T} \right], \end{matrix}$$

where $\mathbf{I}$ is a $p\times p$ identity matrix and $\mathbf{1}$ is a $p$-dimensional vector of 1’s.

Evaluating $\nabla g(\mathbf{X})$ at $\mathbf{X}=\boldsymbol{\mu}_{X}$, substituting back into the variance approximation, letting $\omega=\frac{\boldsymbol{\mu}_{X}}{\sum_{j=1}^{p} \mu_{X,j}}$ gives equation (2) in the text.
