# Supplementary material for: A Bayesian method for detecting pairwise associations in compositional data
Source: PLoS Comput Biol. 2017 Nov 15;13(11):e1005852. doi: 10.1371/journal.pcbi.1005852 (PMC5706738; doi:10.1371/journal.pcbi.1005852)
Supplement: S2 Text — Descriptions of how the datasets were generated for both the challenging scenarios case and for the realistic data case. (DOCX) [file pcbi.1005852.s002.docx]

Detailed Description of Simulated Datasets

# Small Datasets for Challenging Scenarios

Four challenging scenarios tested BAnOCC’s performance on “edge cases” with strong spurious correlations. Given a fixed correlation matrix ($\mathbf{R}_{\log\mathbf{X}}$), the log-basis means ($m_{j}$) and standard deviations ($s_{j}$) were sampled from scenario-specific parameter generating distributions. Nine features and 1,000 samples formed each dataset; the small number of features made the correlation difficult to infer in all cases. Two scenarios were “null” in having no true correlations, while two were “spiked” in having at least one true correlation in the unconstrained counts.

No true correlations were present for two of the scenarios, which differed by the presence of a negative-dominant type spurious correlation. The “simple” scenario had parameter generating distributions with small variance (**1 Fig**) which meant that the spurious correlations present were due primarily to the small number of features. The “high spurious” scenario had a strong negative-dominant type correlation induced by combining a parameter generating distribution for $m_{j}$ with small mean and variance with a parameter generating distribution for $s_{j}$ with large mean and variance (**2 Fig**). This meant that the unconstrained count means ($\mu_{X,j}=e^{m_{j}+0.5s_{j}^{2}}$) and variances ($\sigma_{X,j}^{2}=\mu_{X,j}^{2}(e^{s_{j}^{2}-1})$) were determined by the value of $s_{j}$ so that features with high $\mu_{X,j}$ would also have large $\sigma_{X,j}^{2}$, inducing a negative-dominant type spurious correlation.

Several true correlations were present in the remaining scenarios, which were again distinguished by the presence or absence of a negative-dominant type spurious correlation. The “retained spike” scenario had the same parameter generating distributions as the “simple” scenario (**3 Fig**), while the “reversed spike” scenario had the same parameter generating distributions as the “high spurious” scenario (**4 Fig**). Because the spurious correlations in the “retained spike” scenario were due primarily to the small numbers of features, the magnitude and direction of the true associations were approximately retained between the log-basis and the composition. The features in the “reversed spike” scenario were ordered such that the negative dominant spurious correlation was between two features with a positive true correlation; this caused the correlation to reverse direction when comparing the log-basis and the composition.

# Realistic Data for Performance Comparison

To understand how BAnOCC performs in practice and would compare with other methods, we used more realistic simulated data generated by the sparseDOSSA software, version 1.1.0 [1], which generates synthetic data that closely resemble datasets from microbial ecology. Briefly, it models each feature as a zero-inflated, truncated log-normal distribution with subsequent rounding and estimates the feature-specific parameters using a template dataset. This generative model differs from our assumed model by the incorporation of truncation, zero inflation, and rounding. These differences make the simulated data more realistic and also allow us to assess the robustness of BAnOCC to model violations.

We induced correlations between the features by setting off-diagonal elements of $\mathbf{R}_{\log\mathbf{X}}$, the log-basis correlation, to non-zero values. By default, sparseDOSSA assumes that the features are uncorrelated. We added at most $p/2$ correlations by randomly selecting several elements of $\mathbf{R}_{\log\mathbf{X}}$ to be non-zero. The apparent value of the correlation in the simulated data is somewhat attenuated from the specified value due to the truncation, zero inflation, and rounding. All correlations were given the same value, and we used four different correlation strengths, $\{-0.7,-0.3,0.3,0.7\}$, to compare performance on positive or negative, weak or strong correlations.

For SparseDOSSA calibration, we chose two template datasets: a vaginal dataset from the Human Microbiome Project (HMP) [2] with generally low ecological diversity (i.e. few abundant features per sample), and a stool dataset from the HMP with higher diversity (i.e. more abundant, even features per samples). After an initial filtering, the former (vaginal model) dataset contained 14 features, and the latter (stool model) contained 89. The number of correlations spiked in depended on the template dataset: for the small template with 14 features, we added seven associations, while for the large template with 89 features, we added 15 associations.

For each template and true correlation strength, we simulated 105 datasets with 100 samples and 14 (low diversity) or 89 (high diversity) features. The 105 datasets gave a slight buffer should any method fail on a particular dataset. Before running any of the methods, we removed features which were entirely zero because they could be considered effectively absent for that particular dataset. Type I and type II error rates were calculated only if at least 100 tests were performed for a given template, true correlation strength, and method.

We performed additional simulations with differing numbers of samples (50, 100, or 150) using the two template datasets (high-diversity with 89 features or low-diversity with 14 features) and four correlation strengths (-0.7,-0.3,0.3,0.7). For each template, the pairs of associated features were the same across the sample sizes and correlation strengths.


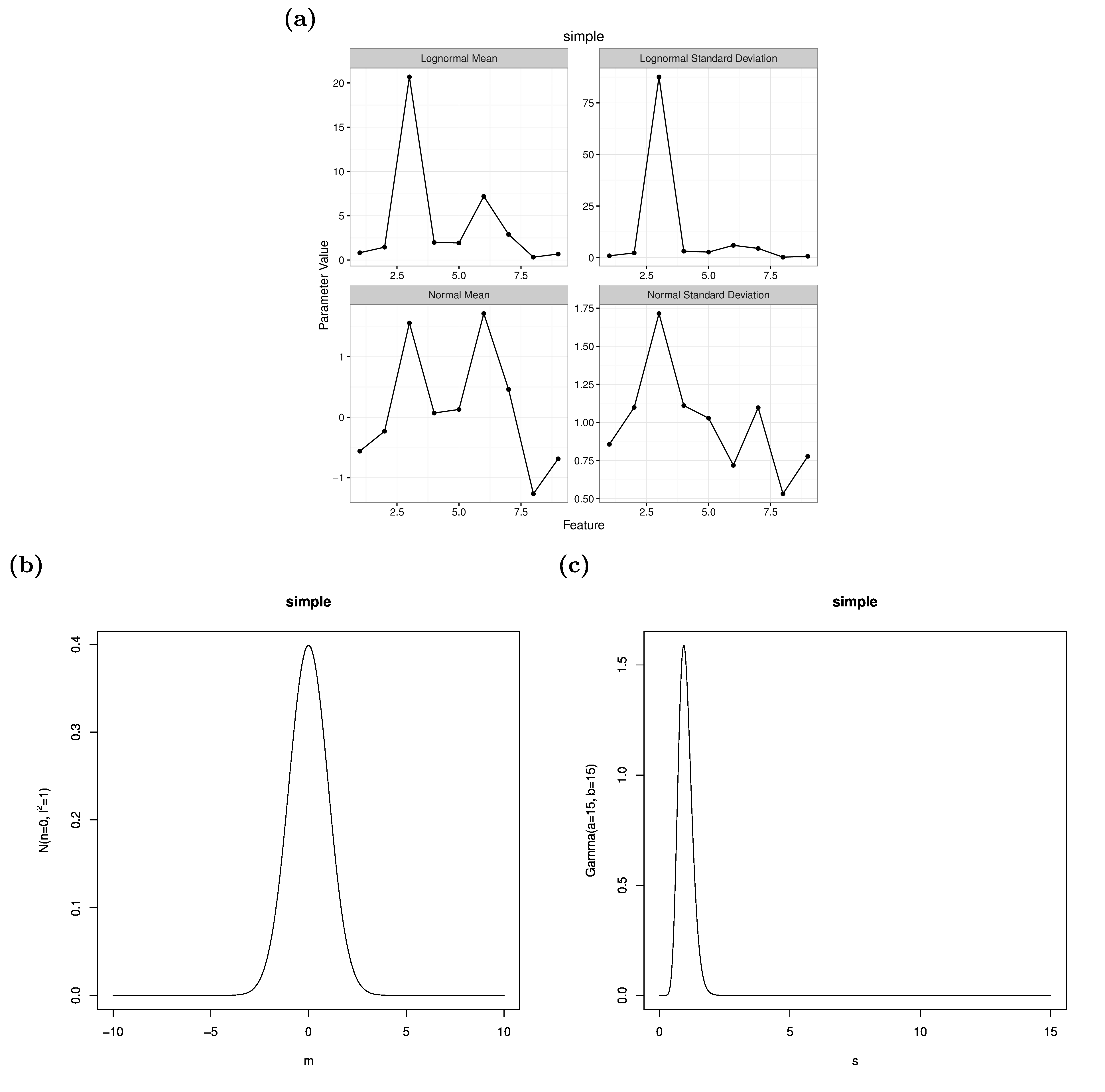


**Fig 1. Parameters and parameter-generating distributions for the “simple” simulation scenario.** The parameters for the “simple” scenario were pretty similar to each other on the normal scale (**A**) because the parameter-generating distributions had small variance (**C**-**D**). This meant that if the number of features was large, the spurious correlation should not be very strong.


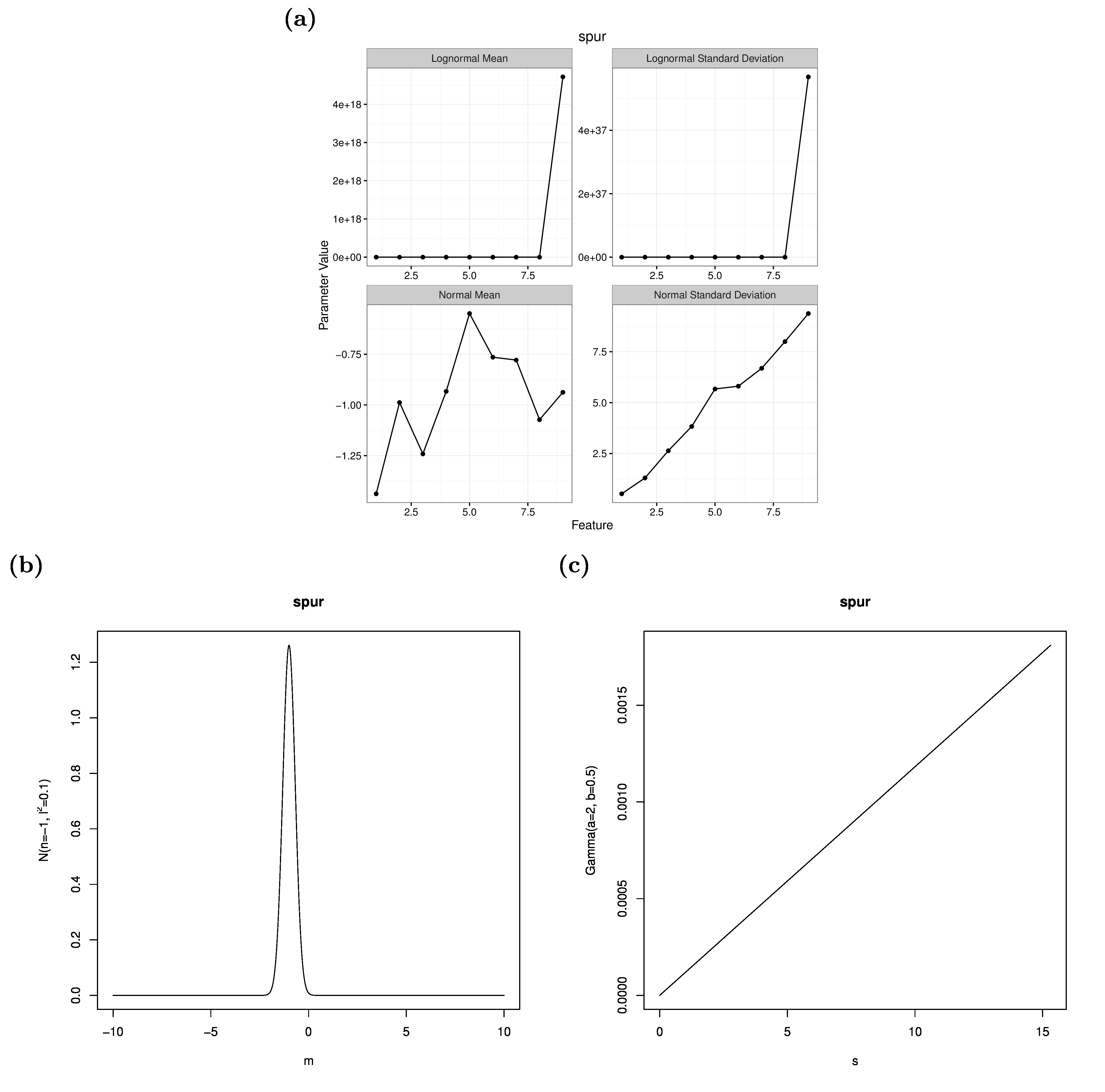


**Fig 2. Parameters and parameter-generating distributions for the “high spurious” simulation scenario.** The “high spurious” scenario had a negative dominant correlation resulting from a positive relationship between the unconstrained count (lognormal) means and variances (**A**). This resulted from a parameter-generating distribution for $m_{j}$ with small mean and variance combined with a parameter-generating distribution for $s_{j}$ with large mean and variance (**B**-**C**). This implied that the unconstrained count mean ($\mu_{X,j}=e^{m_{j}+\frac{1}{2}s_{j}^{2}}$) and variance ($\sigma_{X,j}=\mu_{X,j}^{2}(e^{s_{j}^{2}}-1)$) were determined by the value of $s_{j}$.


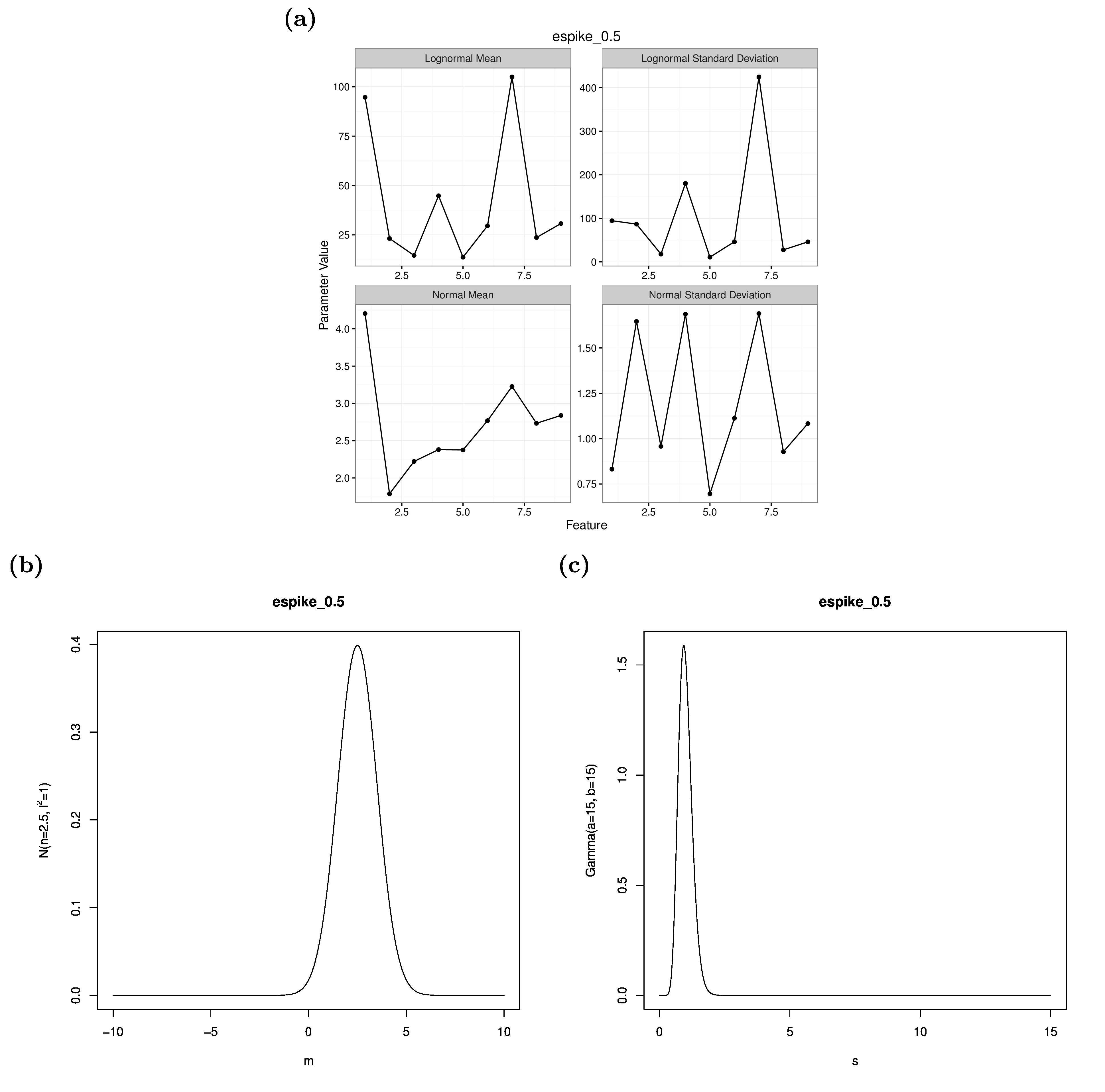


**Fig 3. Parameters and parameter-generating distributions for the “retained spike” simulation scenario.** The features in the “retained spike” scenario had parameter values very similar to each other (**A**) as a result of the small variance of the parameter-generating distributions (**C**-**D**).


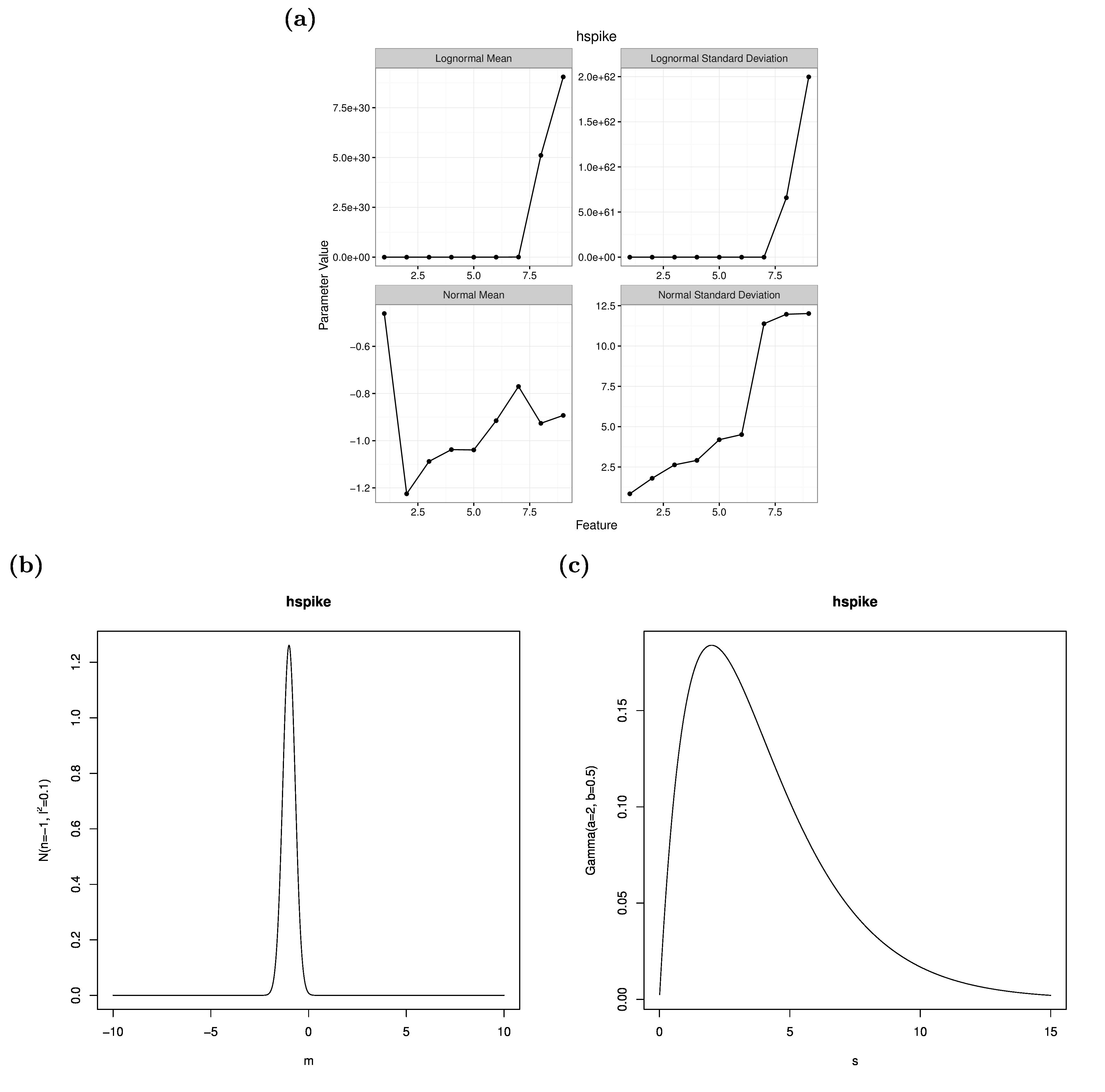


**Fig 4. Parameters and parameter-generating distributions for the “reversed spike” simulation scenario.** The “reversed spike” scenario had a negative dominant spurious correlation introduced due to the fact that the two features with the highest mean also had the highest variance (**A**). This resulted from very low variance in the log-basis mean ($m_{j}$) generating distribution but high variance in the log-basis standard deviation ($s_{j}$) generating distribution (**B**-**C**). This implied that the log-basis mean ($\mu_{X,j}=e^{m_{j}+\frac{1}{2}s_{j}^{2}}$) and log-basis variance ($\sigma_{X,j}=\mu_{X,j}^{2}(e^{s_{j}^{2}}-1)$) were determined primarily by the value of $s_{j}$ and therefore positively correlated.

# References

1. Ren B, Schwager E, Tickle TL, Huttenhower C. sparseDOSSA: Sparse data observations for simulating synthetic abundance. 2016.

2. The Human Microbiome Consortium. Structure, function and diversity of the healthy human microbiome. Nature. 2012;486: 207–214.
