# Supplementary material for: A Bayesian method for detecting pairwise associations in compositional data
Source: PLoS Comput Biol. 2017 Nov 15;13(11):e1005852. doi: 10.1371/journal.pcbi.1005852 (PMC5706738; doi:10.1371/journal.pcbi.1005852)
Supplement: S3 Text — Details on how each of the methods compared in the Results section were implemented, run on the simulated data, and evaluated for type I and type II errors. (DOCX) [file pcbi.1005852.s003.docx]

Implementation of Methods Compared

It is important to remember that H_0_: $\rho_{X,jk}=0$ is equivalent to H_0_: $\rho_{\log X,jk}=0$ when $\mathbf{X}$ follow a log-normal distribution.

# Simplicial Variation

Simplicial variation [1] is based on the variance of logrations. Specifically, the statistic comparing features $j$ and $k$ is the variance of log-ratios

$$t_{jk}=\frac{1}{n-1}\sum_{i=1}^{n} \left( \log\frac{c_{ij}}{c_{ik}}-\overline{\log\frac{c_{ij}}{c_{ik}}} \right)^{2}=\frac{1}{n-1}\sum_{i=1}^{n} \left( \log\frac{x_{ij}}{x_{ik}}-\overline{\log\frac{x_{ij}}{x_{ik}}} \right)^{2}$$

It can be seen that $t_{jk}$ will be zero if $X_{ij}\propto X_{ik}$ for $i=1,\ldots,n$, and in this case features $j$ and $k$ would be perfectly correlated.

To test whether the correlation between the unobserved counts of features $j$ and $k$ ($\rho_{X,jk}$) was zero, we used a one-sided permutation test on $\frac{1}{t_{jk}}$, which should be large if $X_{j}$ and $X_{k}$ are correlated. This test is approximate, as after sample permutation, $\frac{c_{i_{1}j}}{c_{i_{2}k}}$ is not necessarily equal to $\frac{x_{i_{1}j}}{x_{i_{2}k}}$ .

The permutation p-value was used to determine type I and type II error rates. A positive result was obtained if the p-value was less than the test level ($0.05$), and these were compared with the true zero or non-zero values of the correlation matrix.

# sparCC

sparCC [2] relies on the simplicial variation statistic, but uses an assumption about $\sum_{i=1}^{p} \sum_{j=i+1}^{p} w_{jk}$ to impose sparsity and estimate $w_{jk}$ through a system of iteration.

We used the authors’ software from <https://bitbucket/yonatanf/sparcc>, downloaded on December 14, 2015. The authors propose a bootstrap based method for inference, and we used this method with the software’s default parameters of 100 bootstrap datasets and two-sided p-values. The type I and type II errors were determined based on the correct or incorrect rejection of H_0_: $w_{jk}=0$ where rejection was determined by a two-sided p-value of less than $0.05$.

# CCLasso

CCLasso [3] uses a LASSO penalty on the off-diagonal elements of $\mathbf{R}_{\log\mathbf{X}}^{-1}$ with a loss function of

$$\mathrm{LOSS}(\mathbf{R}_{\log\mathbf{X}})=\frac{1}{2}\mathrm{tr}\left[ \left\{ \mathbf{G}\left( \mathbf{R}_{\log\mathbf{X}}-\mathbf{R}_{C} \right)\mathbf{G}^{T} \right\}diag\left( \mathbf{G}\mathbf{R}_{C}\mathbf{G}^{T} \right)^{-1}\left\{ \mathbf{G}\left( \mathbf{R}_{\log\mathbf{X}}-\mathbf{R}_{C} \right)\mathbf{G}^{T} \right\} \right],$$

where $\mathbf{G}=\mathbf{I}-\frac{1}{p}\mathbf{1}\mathbf{1}^{T}$, which corresponds to a centered log-ratio transformation of $\mathbf{C}_{i}$. The tuning parameter $\lambda$ is chosen using $k$-fold cross validation. We downloaded the authors’ software from <https://github.com/huayingfang/CCLasso> on December 10, 2015. We used the default parameters of 3-fold cross-validation, a tuning parameter interval of $[0.0001,1]$, and a maximum number of selection iterations of 20.

Because CCLasso is LASSO-based, there is no accompanying inference method. We determined type I and type II errors as the authors do in their paper: by correct or incorrect estimation of $w_{jk}$ to be exactly zero. That is, a positive result was $w_{jk}$ estimated to be non-zero.

# SPIEC-EASI

SPIEC-EASI [4] estimates $\mathbf{R}_{\log\mathbf{X}}^{-1}$ using either the neighborhood selection method of [5], or the graphical LASSO method of [6]. The tuning parameter selection is done using stability selection via the StARS algorithm of [7]. We downloaded the authors’ software from <https://github.com/zdk123/SpiecEasi> on June 7, 2016. We used a minimum lambda ratio of $0.01$, 500 subsamplings for stability selection, and 100 lambda values.

SPIEC-EASI is also LASSO-based, and thus provided no inference method. As for CCLasso (above), we determined type I and type II errors by correct or incorrect estimation of $w_{jk}$ to be exactly zero. Our simulated datasets were structured such that if $w_{jk}$ was zero, $w_{jk}^{-1}$ would also be zero, so this approach was appropriate.

# ReBoot

ReBoot is a permutation-based approach to test arbitrary similarity measures in the presence of compositionality [8]. We used the R package CCREPE, which implements with permutation approach, version 1.6.0 [9]. The method involves specifying the similarity measure as well as the number of iterations. We used Spearman correlation as the similarity measure, and 1,000 iterations. The package also implements some zero filtering, and we used the default of excluding features with more than ${0.001}^{\frac{1}{n}}$ zeros from analysis.

ReBoot provides a p-value for H_0_: $\rho_{X,jk}=0$, and we used correct or incorrect rejection of H_0_ to determine type I and type II errors.

# Spearman Correlation

Spearman correlation served as a “naive” estimate of $\mathbf{R}_{X}$ that does not take into account the compositional structure of the data. We used a two-sided permutation test with 1000 permutations to evaluate significance, and determined type I and type II errors by correct or incorrect rejection of H_0_: $\rho_{X,jk}=0$.

We applied Spearman correlation twice: once on the simulated unconstrained counts (which, of course, are unobserved in practice) and once on the simulated compositions. The former should have controlled type I error rates and good power because Spearman correlation would be appropriate if the data are unconstrained; the latter should have very high type I error rates because the compositions are constrained.

# BAnOCC

BAnOCC samples the posterior using No-U-Turn Sampling (NUTS) [10], a Hamiltonian Monte Carlo (HMC)-based algorithm, as provided in the rstan R package [11]. For all datasets, both simulated and real we started with 1000 iterations of warmup (necessary to choose the appropriate step size for HMC), and 4000 iterations of sampling for each of at least three chains. We evaluated convergence of the chains using the R-hat statistic [12] and increased the number of iterations until the R-hat statistics for all sampled parameters were less than 1.1.

1. Aitchison J. A concise guide to compositional data analysis. 2nd compositional data analysis workshop. 2003.

2. Friedman J, Alm EJ. Inferring correlation networks from genomic survey data. PLoS Comput Biol. 2012;8: e1002687.

3. Fang H, Huang C, Zhao H, Deng M. CCLasso: Correlation inference for compositional data through lasso. Bioinformatics. 2015;31: 3172–3180.

4. Kurtz ZD, Müller CL, Miraldi ER, Littman DR, Blaser MJ, Bonneau RA. Sparse and compositionally robust inference of microbial ecological networks. PLoS Comput Biol. 2015;11: 1–25.

5. Meinshausen N, Bühlmann P. High-dimensional graphs and variable selection with the lasso. The Annals of Statistics. 2006;34: 1436–1462.

6. Friedman J, Hastie T, Tibshirani R. Sparse inverse covariance estimation with the graphical lasso. Biostatistics (Oxford, England). 2008;9: 432–441.

7. Liu H, Roeder K, Wasserman L. Stability approach to regularization selection (stARS) for high dimensional graphical models. In: Lafferty JD, Williams CKI, Shawe-Taylor J, Zemel RS, Culotta A, editors. Advances in neural information processing systems 23. 2010. pp. 1432–1440.

8. Faust, K. and Sathirapongsasuti, F., Izard J, Segata N, Gevers D, Raes J, Huttenhower C. Microbial co-occurrence relationships in the human microbiome. PLoS Comput Biol. 2012;8: e1002606.

9. Schwager E, Bielski C, Weingart UG. CCREPE: Compositionality Corrected by REnormalization and PErmutation. 2014.

10. Hoffman MD, Gelman A. The No-U-turn Sampler: Adaptively setting path lengths in hamiltonian monte carlo. J Mach Learn Res. 2014;15: 1593–1623.

11. Stan Development Team. RStan: the R interface to Stan, version 2.6.0. 2014.

12. Gelman A, Rubin DB. Inference from iterative simulation using multiple sequences. Statistical Science. 1992;7: 457–472.
