# Supplementary figures and images for: A Bayesian method for detecting pairwise associations in compositional data
Source: PLoS Comput Biol. 2017 Nov 15;13(11):e1005852. doi: 10.1371/journal.pcbi.1005852 (PMC5706738; doi:10.1371/journal.pcbi.1005852)

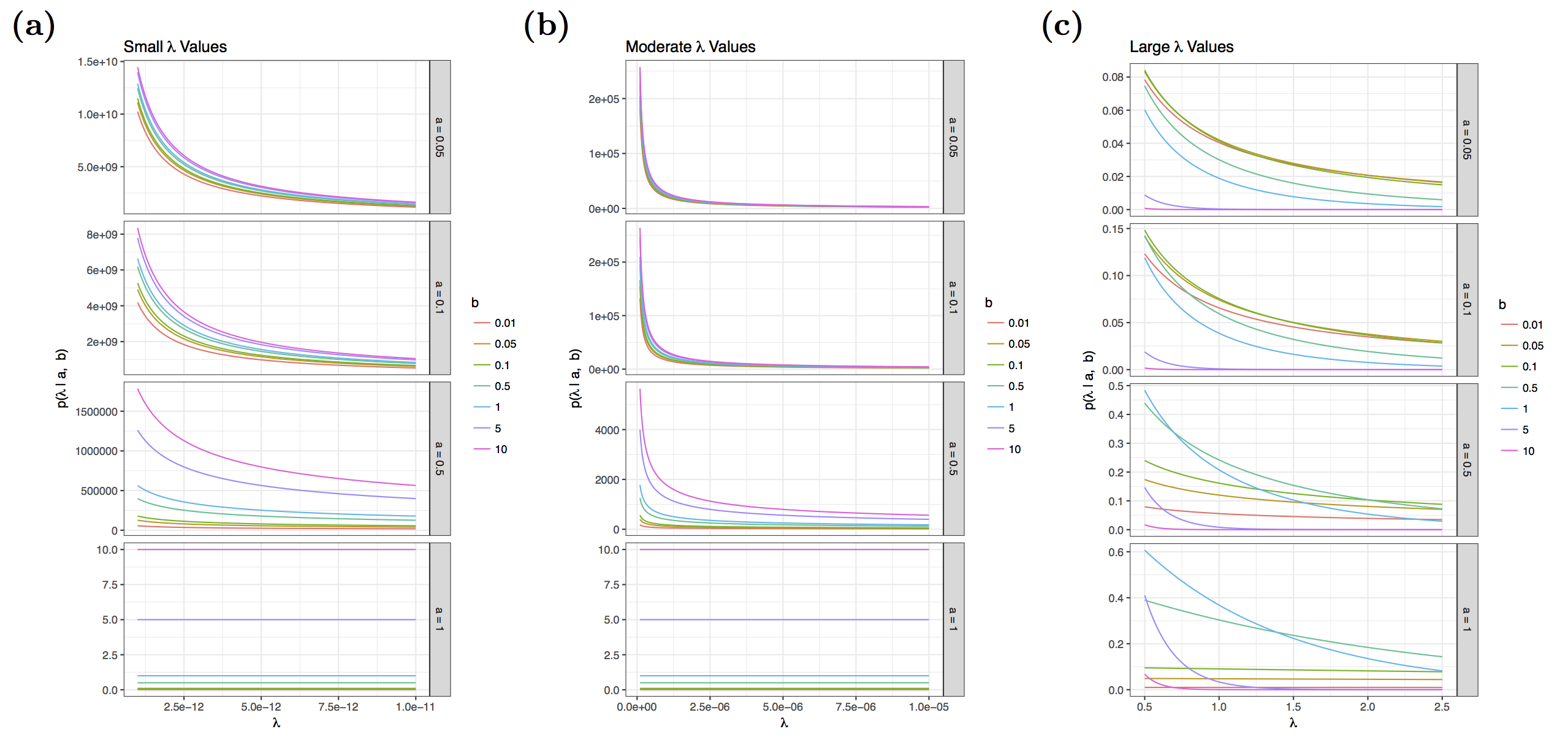

Supplement: S1 Fig — The densities of different priors on λ for different ranges of λ values. The shape parameter a determines how quickly the prior density decreasys, while the rate parameter b determines how much prior weight is placed on small λ values rather than large λ values. (TIF) [file pcbi.1005852.s007.tif]

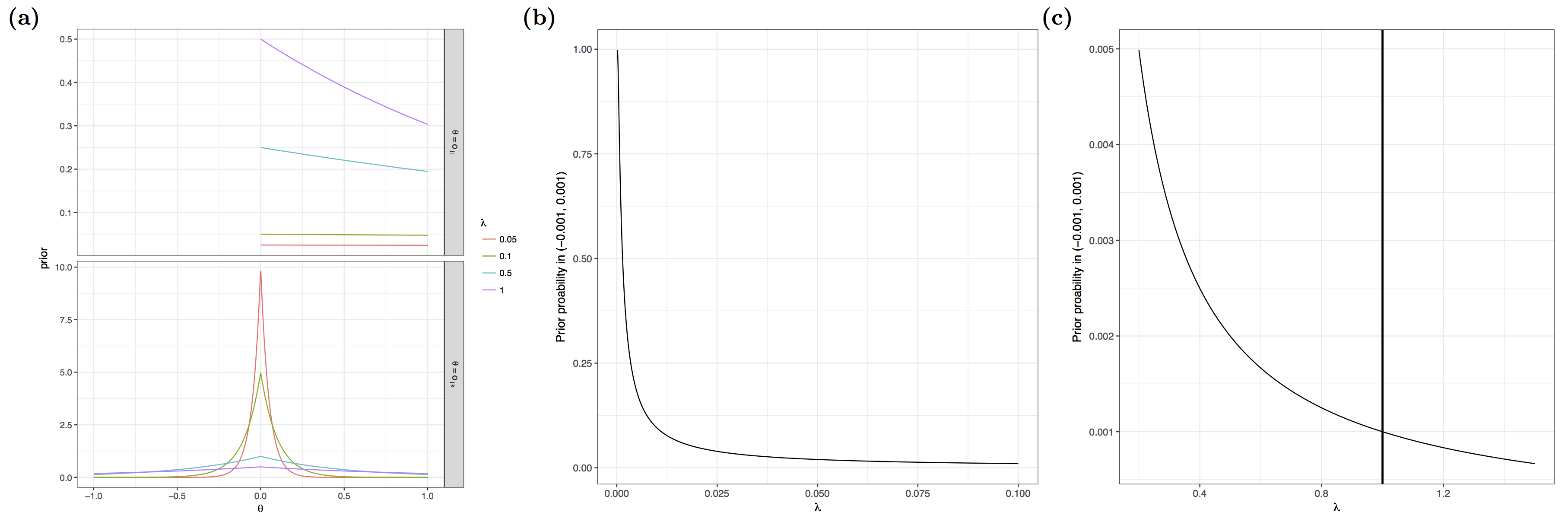

Supplement: S2 Fig — A The shape of the prior on ojk and ojj for several values of λ. Smaller λ results in greater shrinkage towards zero. B-C The prior probability in the interval (−0.001,0.001) for each off-diagonal element ojk| λ∼Laplace(λ) across small (B) or large (C) values of λ. Small values (<0.1) of λ show the greatest shrinkage, while beyond λ = 1 the shrinkage becomes negligible, as shown by the maximal shrinkage for λ > 0.2 being <0.005. (TIF) [file pcbi.1005852.s008.tif]

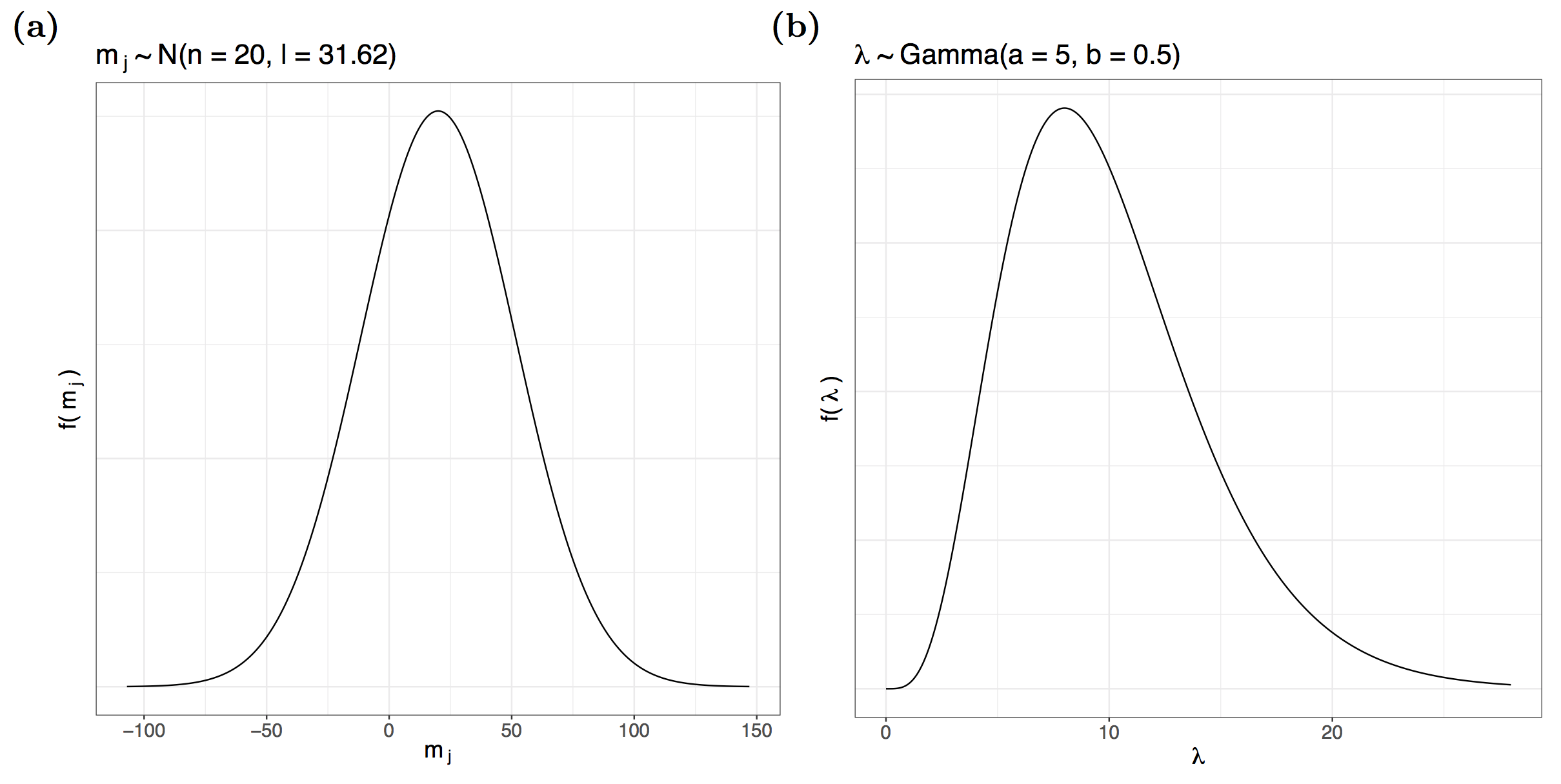

Supplement: S3 Fig — The prior distributions for the test cases used a prior on m that was very uninformative, being centered at 0 and with a large variance. The prior on λ put most prior weight on λ values less than one and had narrow tails to encourage shrinkage of the correlation estimates (B). (TIF) [file pcbi.1005852.s009.tif]

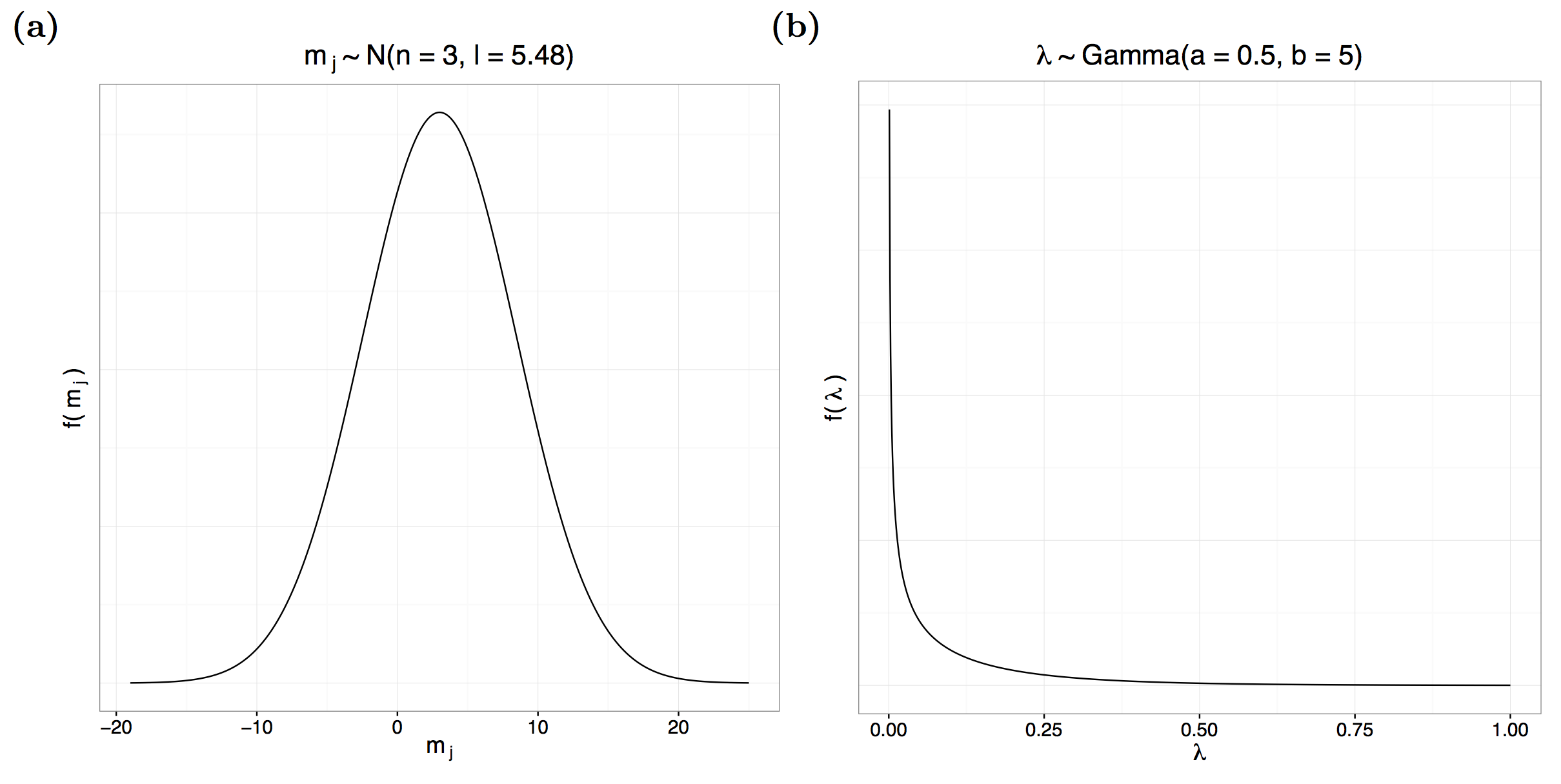

Supplement: S4 Fig — We used a prior for m that gave reasonable behavior for the sum of the unobserved count medians ∑j=114emj (A). The prior on λ put most prior weight on λ values less than one and had narrow tails to encourage shrinkage of the correlation estimates (B). (See also S14 Fig). (TIF) [file pcbi.1005852.s010.tif]

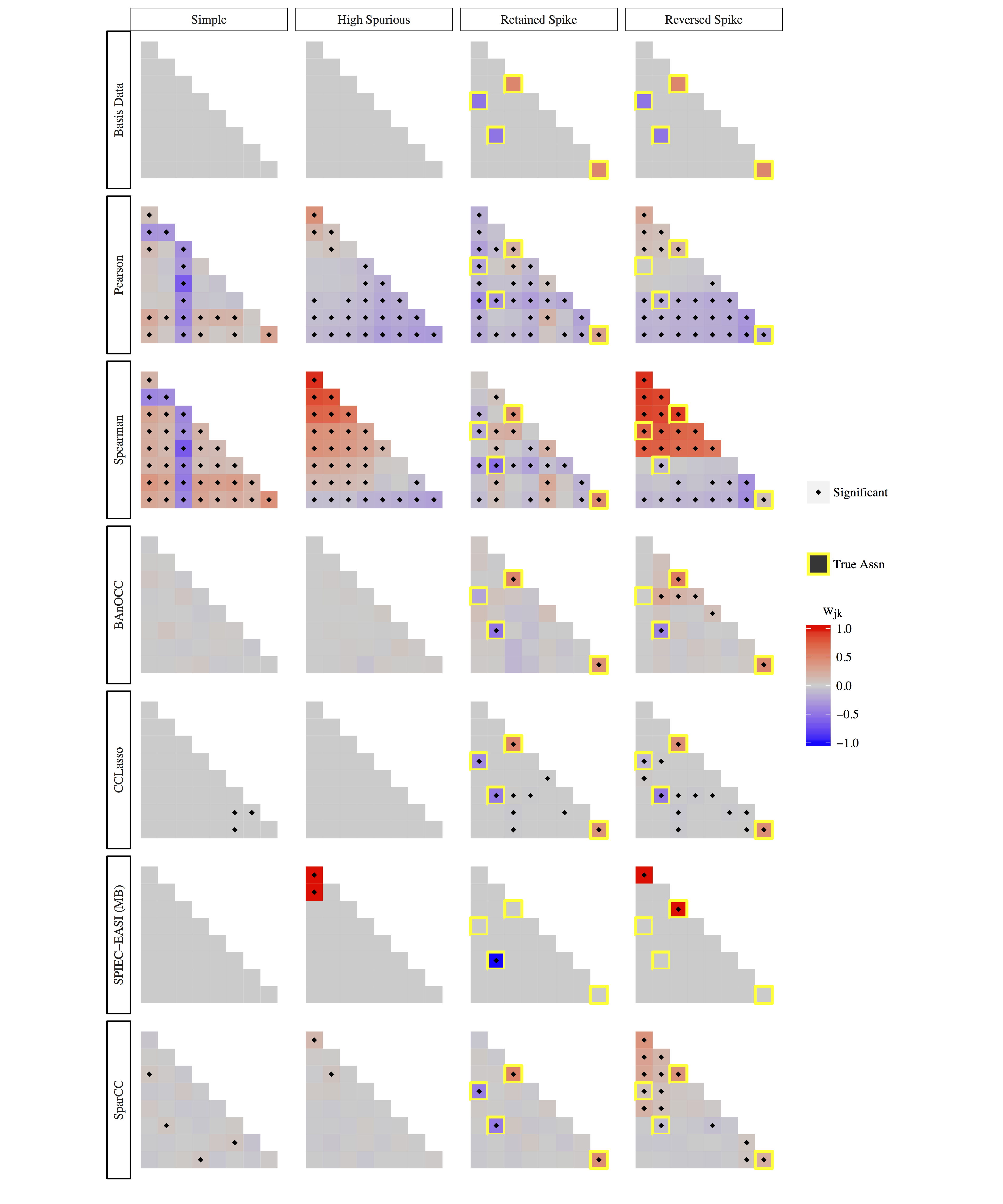

Supplement: S5 Fig — The estimates and significance of several methods on the four scenarios (columns): simple, with no true correlations and no negative dominant spurious correlation; high spurious, with no true correlations and a negative dominant spurious correlation; retained spike, with several true correlations and no negative dominant spurious correlation; and reversed spike, with several true correlations and a negative dominant spurious correlation. The top row is data-derived, with the bottom triangle indicating the true log-basis correlation R^logX and the top triangle the compositional correlation calcualted using the 1,000 samples from the data. BAnOCC evaluates significance using 95 % credible intervals. CCLAsso and SPIEC-EASI (MB) are significant if they are non-zero. SPIEC-EASI (MB) colors indicate the sign rather than the magnitude of the estimated correlations as the estimates are not possible to compute. SparCC evaluates significance using a bootstrap-based method. All the methods do poorly at detecting and correctly estimating the negative correlation between features 1 and 5 in the reversed spike scenario, and instead tend to falsely detect several positive correlations. (TIF) [file pcbi.1005852.s011.tif]

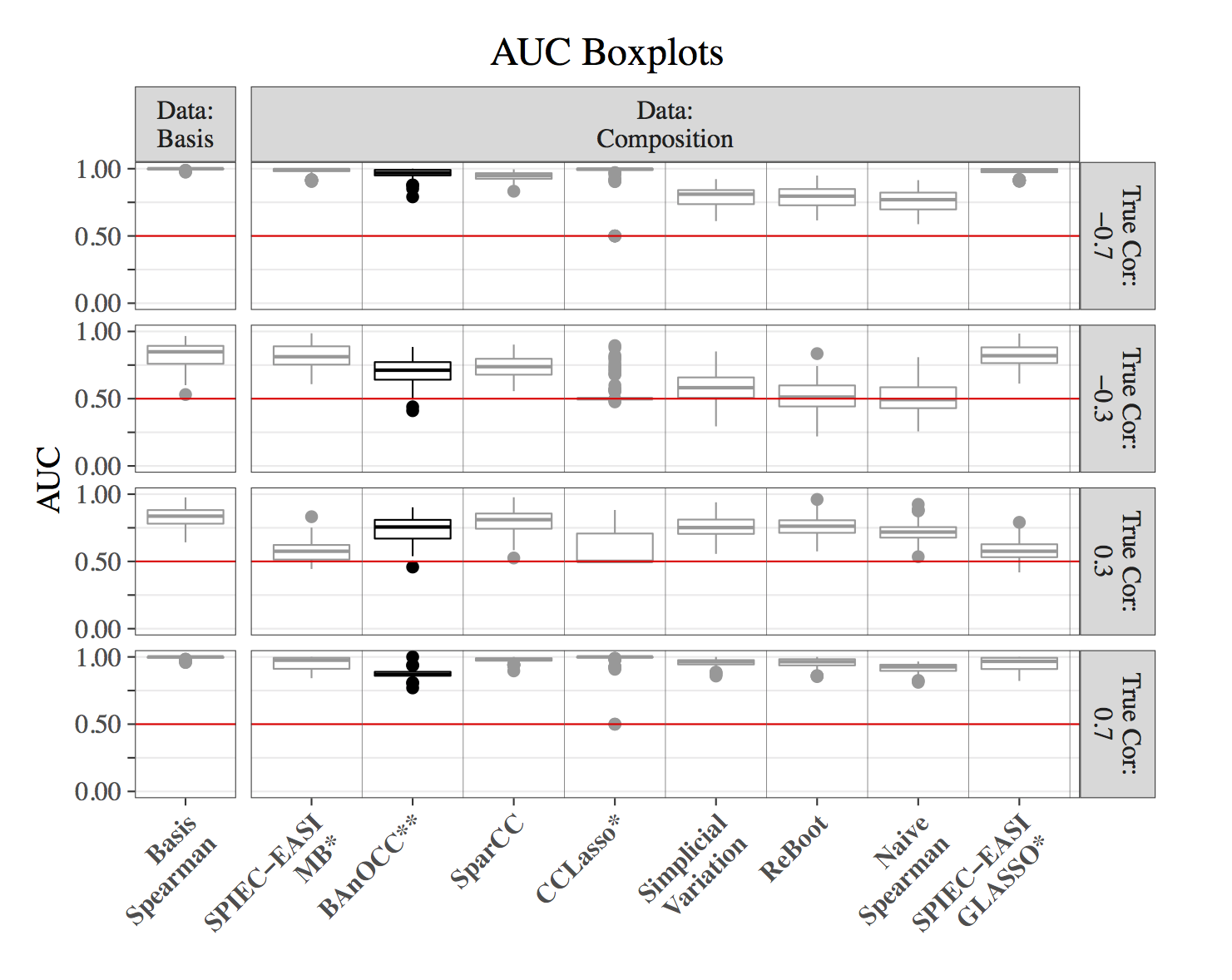

Supplement: S6 Fig — For each given correlation strength and template dataset, AUCs were calculated for each of 105 simulated datasets comprising sparseDOSSA-derived compositions with 100 samples modeled on a low-diversity dataset with 14 features. The ROCs used to measure the AUCs are based on p-values (Spearman correlation, simplicial variation, SparCC), credible intervals (BAnOCC), correlation estimate (CCLasso) or stability score (SPIEC-EASI). Thus each boxplot consists of 105 points. Each of the 105 AUCs are measured over seven true correlations, and all of the methods do better than expected by chance (red line), although BAnOCC has overall the highest average AUC. (TIF) [file pcbi.1005852.s012.tif]

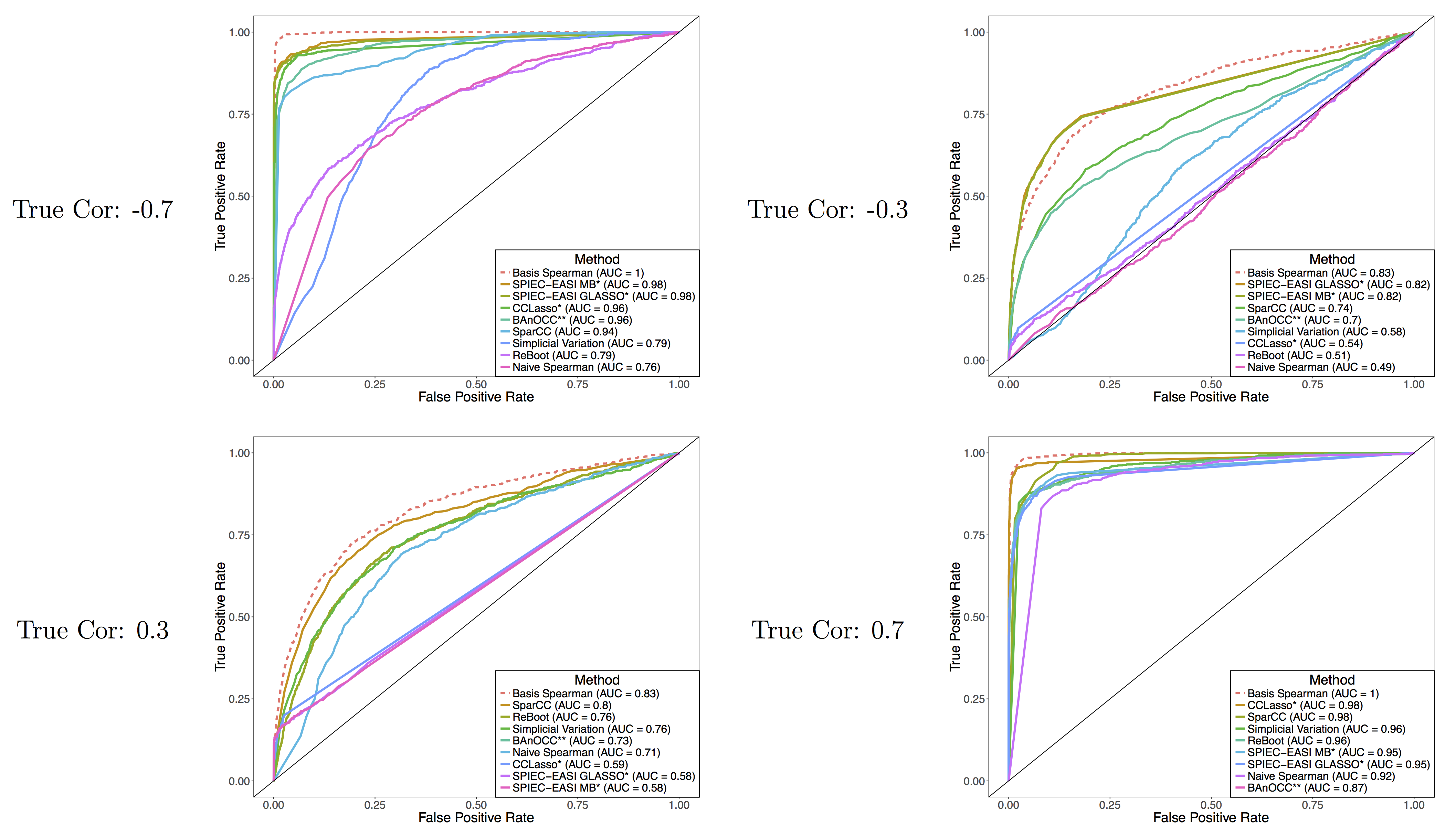

Supplement: S7 Fig — For a given correlation strength, each ROC is calculated over the aggregation of all 735 true associations in 105 simulated datasets comprising SparseDOSSA-derived compositions with 100 samples modeled on a low-diversity dataset with 14 features. The cutoffs used are based on p-values (Spearman correlation, simplicial variation, SparCC), credible interval width (BAnOCC), correlation estimate (CCLasso) or stability score (SPIEC-EASI). (TIF) [file pcbi.1005852.s013.tif]

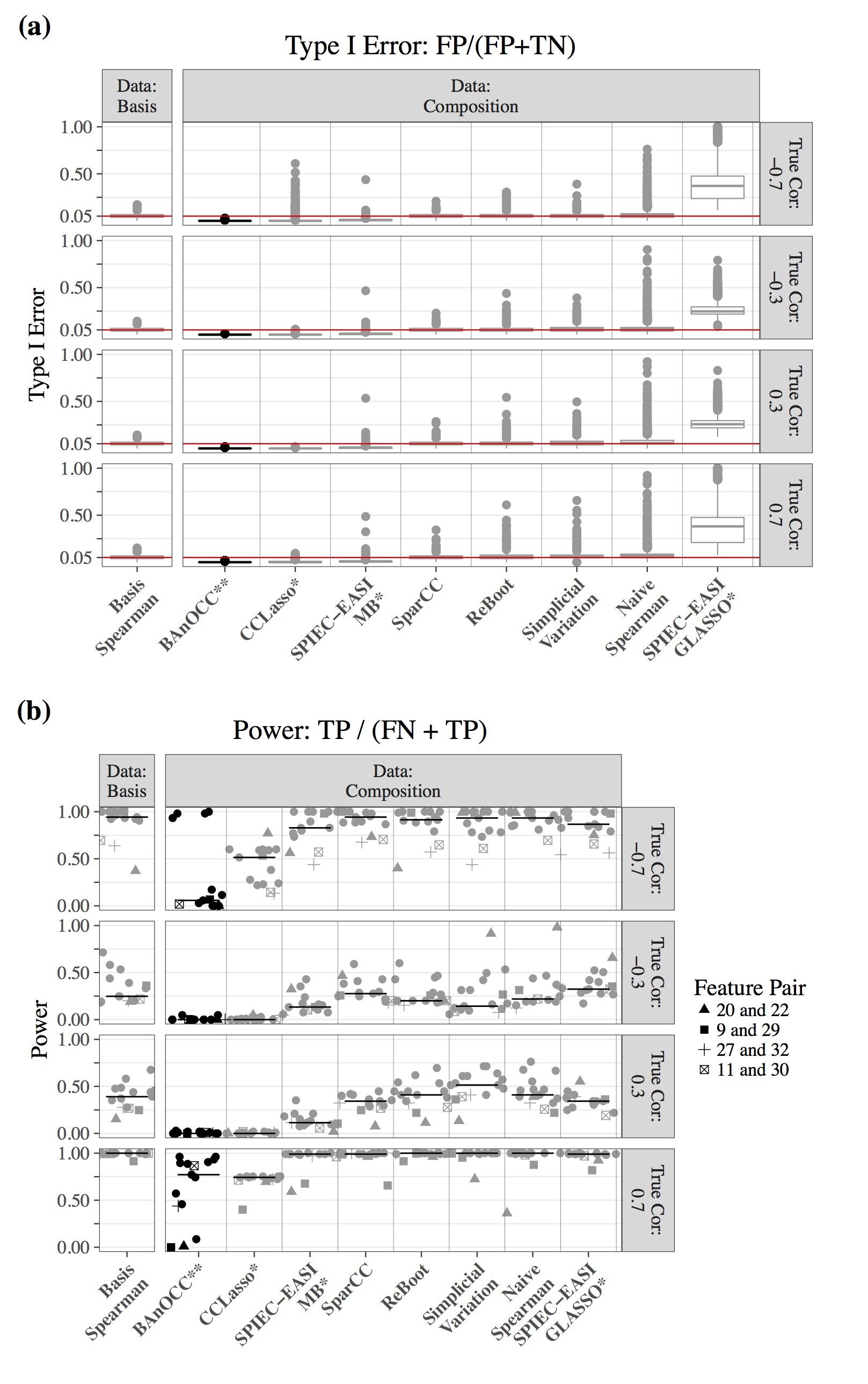

Supplement: S8 Fig — Results on simulated data comprising 100 SparseDOSSA-derived compositions modeled on a high-diversity dataset with 89 features. A Type I error rates are controlled across all correlation values only by BAnOCC. B Power is comparable between BAnOCC and other modern methods across spiked correlation strengths, with BAnOCC and others correctly controlling error rates and only BAnOCC providing full inference and probability distributions on the resulting microbial interaction networks. * = rejection of H0 based on estimation; ** = rejection of H0 based on inference from credible intervals; all others, rejection of H0 based on inference from p-values. (See S16 Fig for the priors used.) (TIF) [file pcbi.1005852.s014.tif]

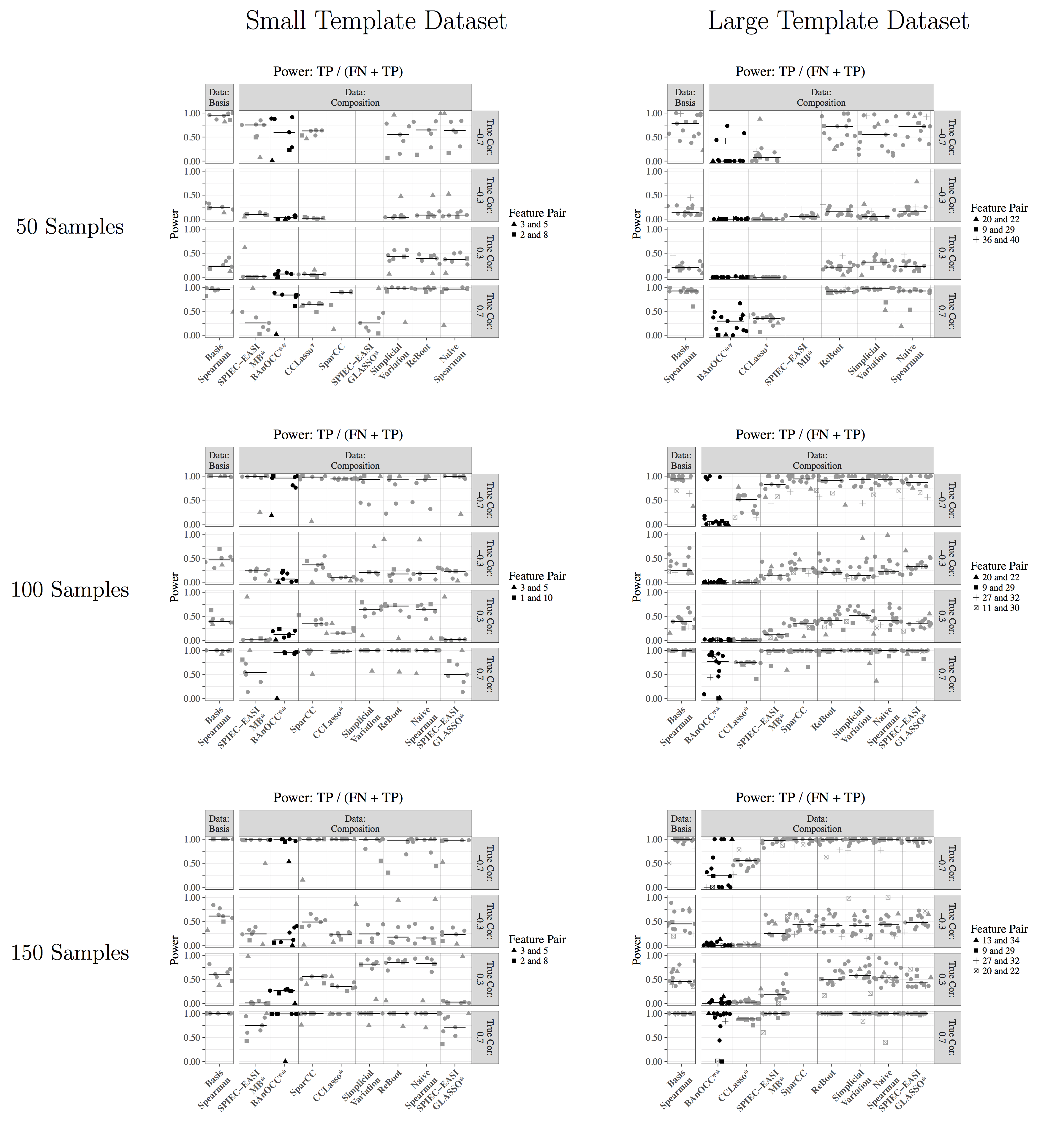

Supplement: S9 Fig — Power on simulated data comprising SparseDOSSA-derived compositions modeled on a low-diversity dataset with 14 features (small template) or a high-diversity dataset with 89 features (large template). See S2 Text for simulation details. The rows correspond to the number of samples (50, 100, or 150) simulated. BAnOCC controls the type I error rate in all scenarios, and the type I error rate behavior for most methods does not change with increasing sample size. * = rejection of H0 based on estimation; ** = rejection of H0 based on inference from credible intervals; all others, rejection of H0 based on inference from p-values. (TIF) [file pcbi.1005852.s015.tif]

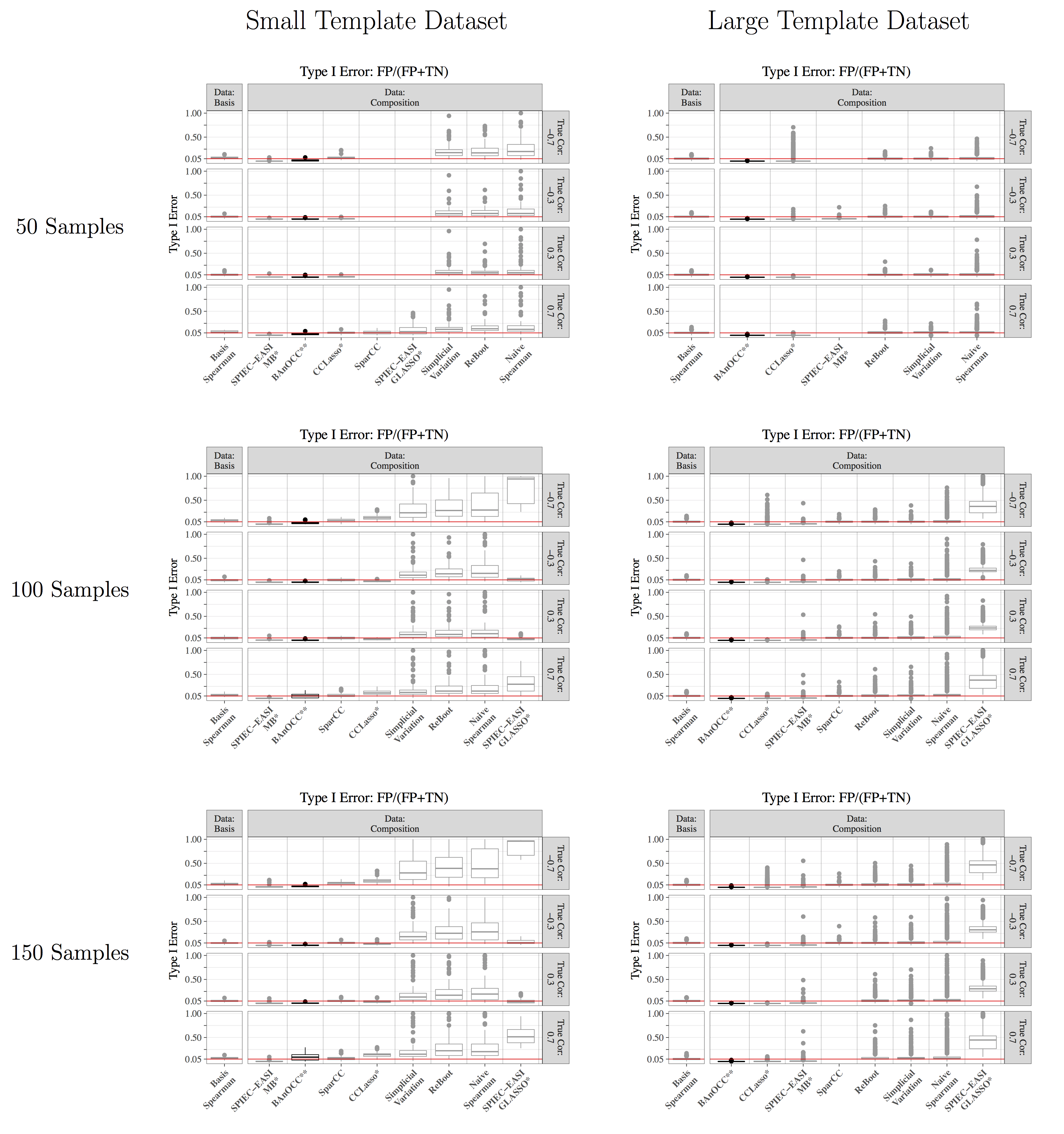

Supplement: S10 Fig — Type I error rates on simulated data comprising SparseDOSSA-derived compositions modeled on a low-diversity dataset with 14 features (small template) or a high-diversity dataset with 89 features (large template). See S2 Text for simulation details. The rows correspond to the number of samples (50, 100, or 150) simulated. BAnOCC controls the type I error rate in all scenarios, and the type I error rate behavior for most methods does not change with increasing sample size. * = rejection of H0 based on estimation; ** = rejection of H0 based on inference from credible intervals; all others, rejection of H0 based on inference from p-values. (TIF) [file pcbi.1005852.s016.tif]

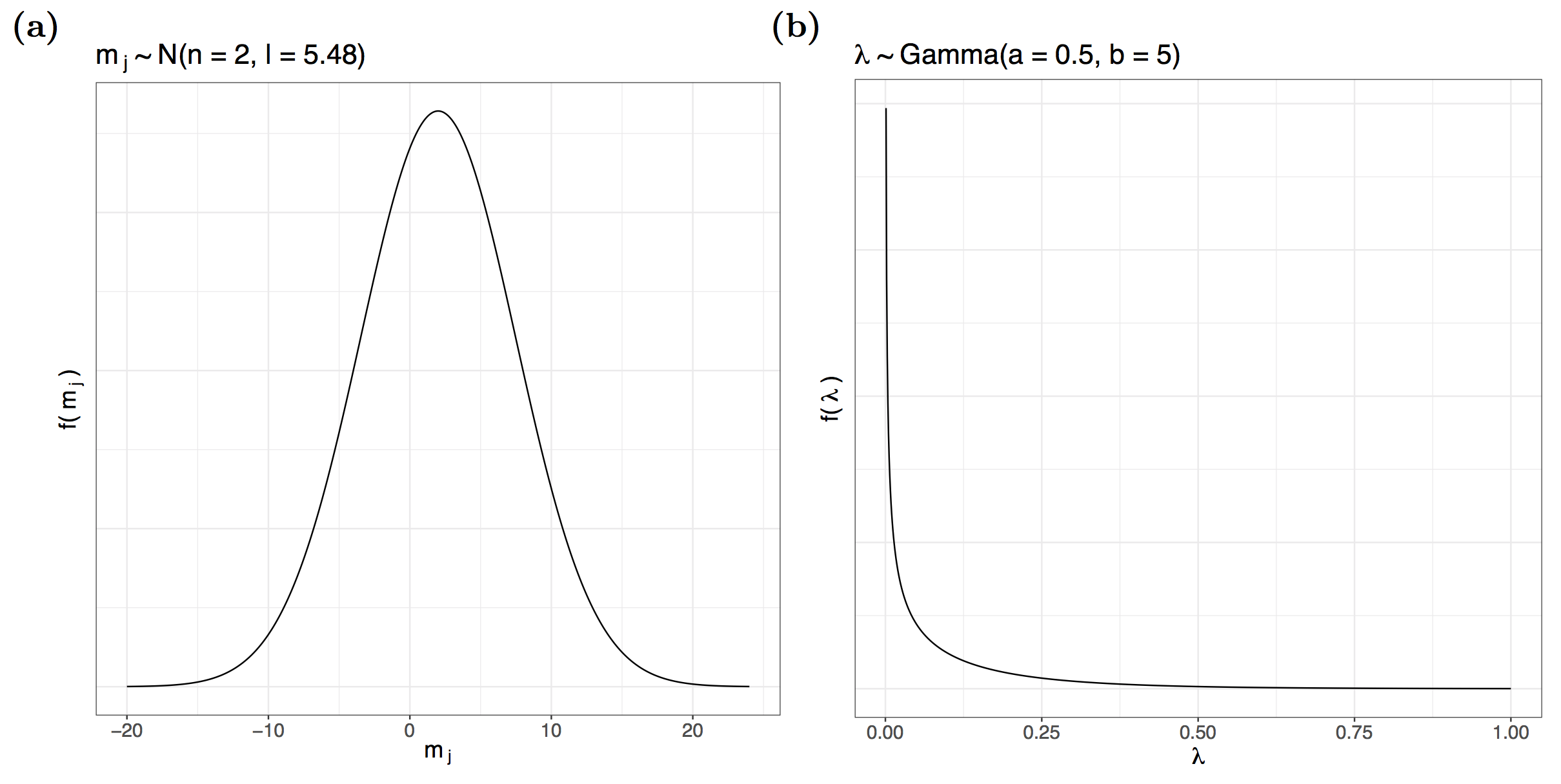

Supplement: S11 Fig — We used a prior for m that gave reasonable behavior for the sum of the unobserved count medians ∑j=124emj (A). The prior on λ put most prior weight on λ values less than one and had narrow tails to encourage shrinkage of the correlation estimates (B). (See also S14 Fig). (TIF) [file pcbi.1005852.s017.tif]

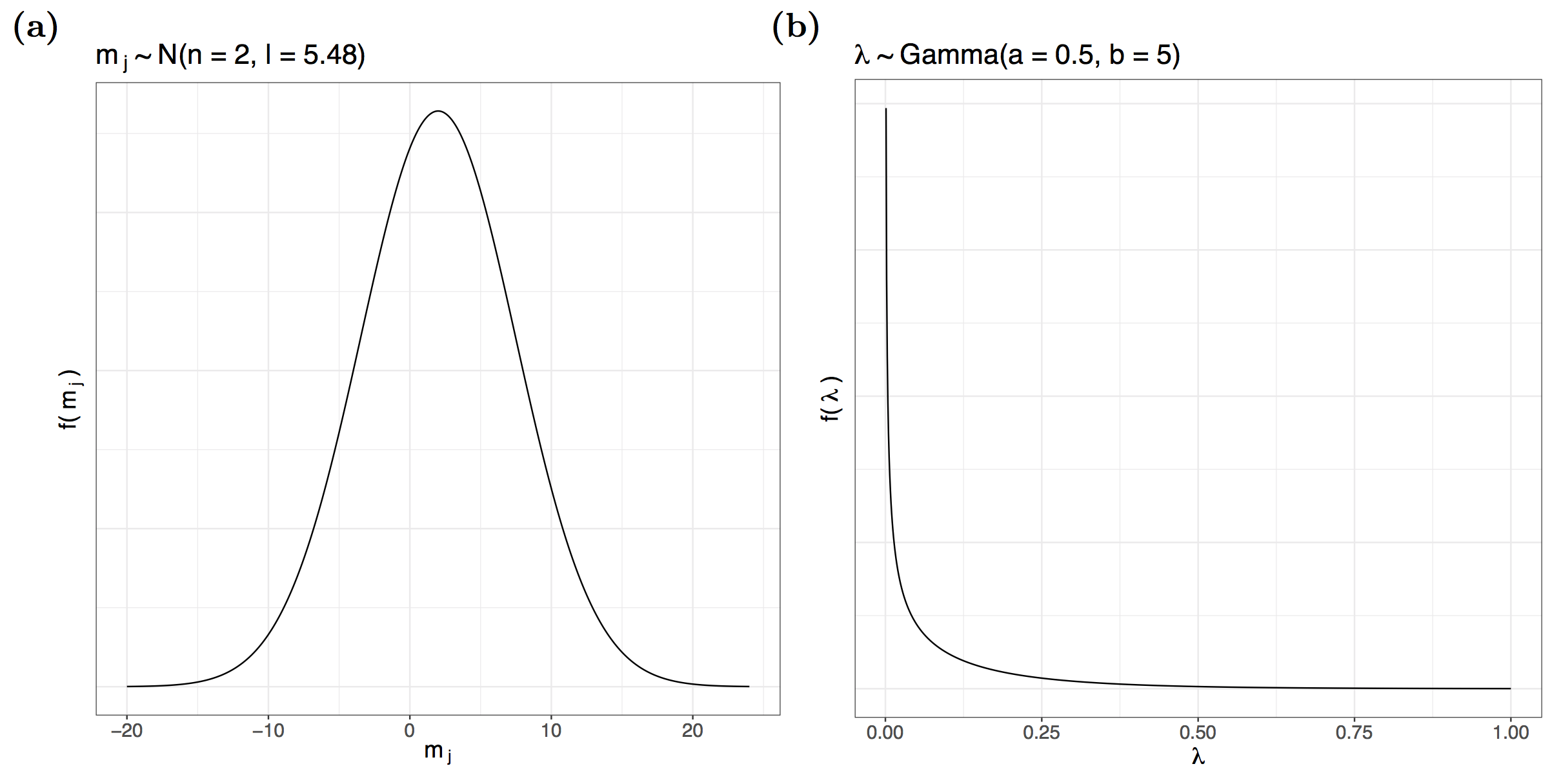

Supplement: S12 Fig — We used a prior for m that gave reasonable behavior for the sum of the unobserved count medians ∑j=121emj (A). The prior on λ put most prior weight on λ values less than one and had narrow tails to encourage shrinkage of the correlation estimates (B). (See also S14 Fig). (TIF) [file pcbi.1005852.s018.tif]

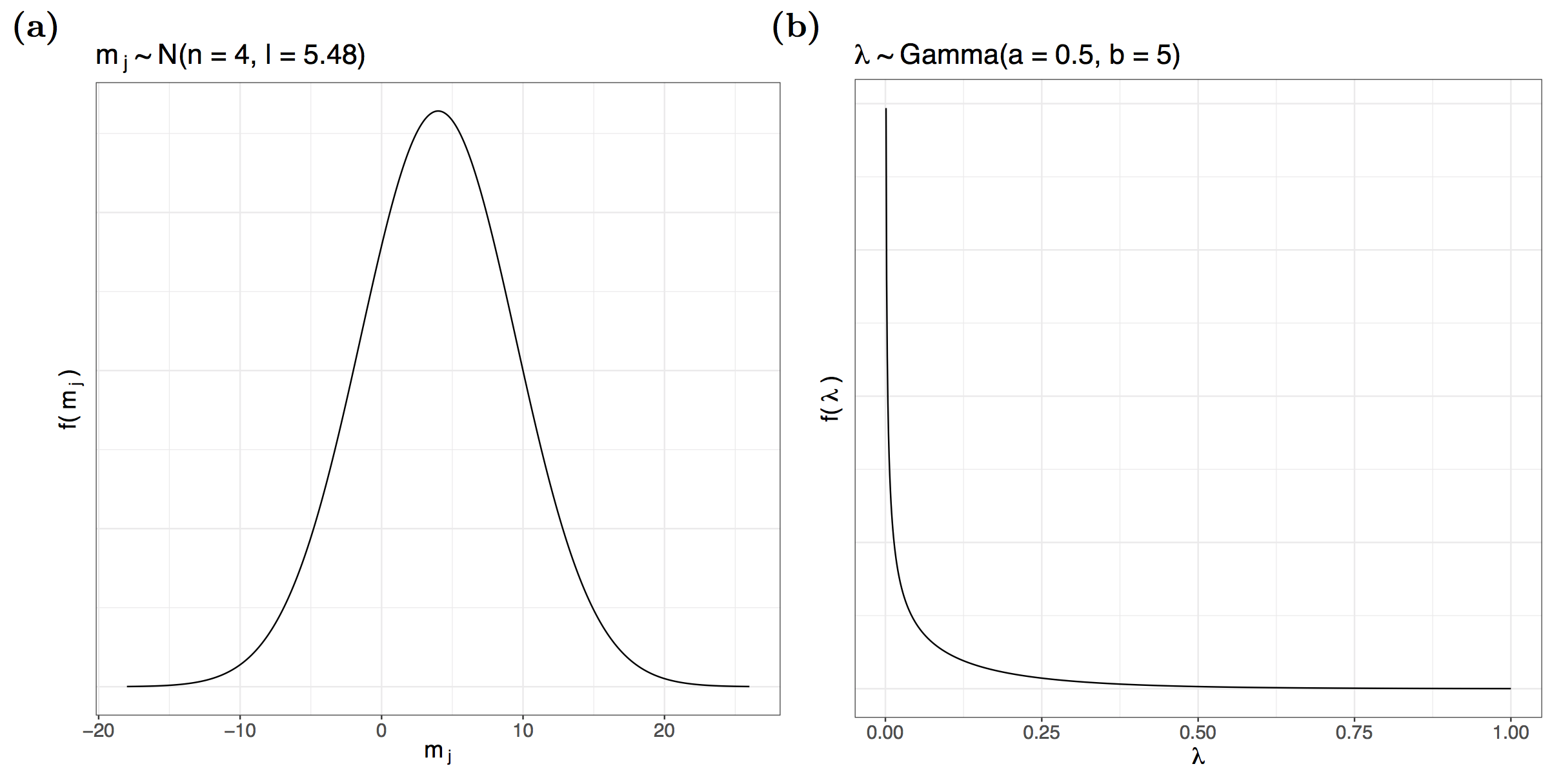

Supplement: S13 Fig — We used a prior for m that gave reasonable behavior for the sum of the unobserved count medians ∑j=111emj (A). The prior on λ put most prior weight on λ values less than one and had narrow tails to encourage shrinkage of the correlation estimates (B). (See also S14 Fig). (TIF) [file pcbi.1005852.s019.tif]

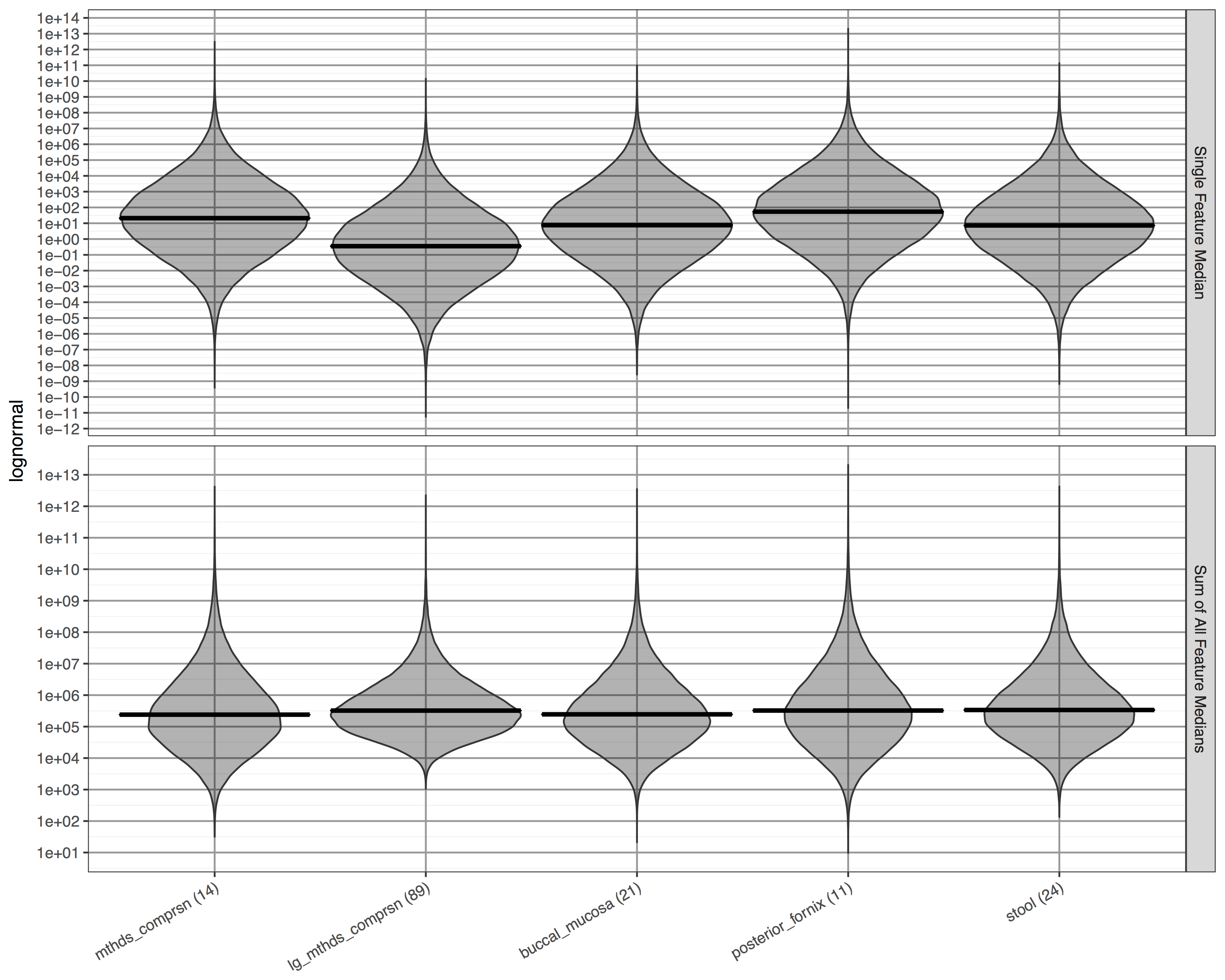

Supplement: S14 Fig — The implied priors on the median unobserved counts emj (top panel) and the sum of the median unobserved counts ∑j=1pemj (bottom panel) for the SparseDOSSA simulated data and the body sites from the application. Each distribution is estimated using 100,000 random samples. The mean of mj was selected such that the sum of the median unobserved counts approximately shared the same average. (TIF) [file pcbi.1005852.s020.tif]

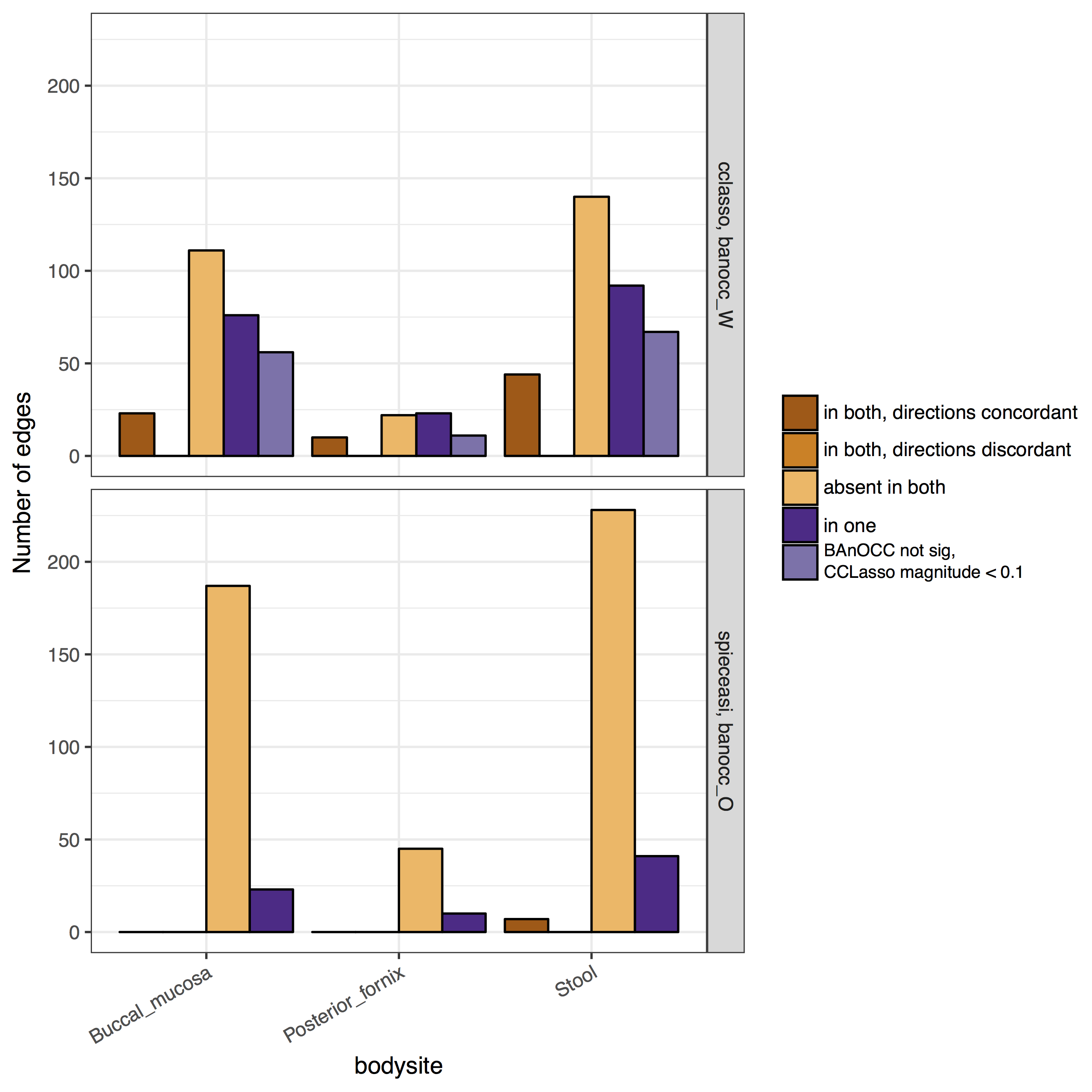

Supplement: S15 Fig — The number of edges significant in both methods, neither method, or only one method, stratified by body site and whether the methods use the log-basis precision or correlation matrix. Most edges are concordantly significant (or not) between both methods; few are significant by only one method. Further, most of the edges significant in CCLasso but not BAnOCC are small in magnitude (BAnOCC not sig, CCLasso magnitude < 0.1). (TIF) [file pcbi.1005852.s021.tif]

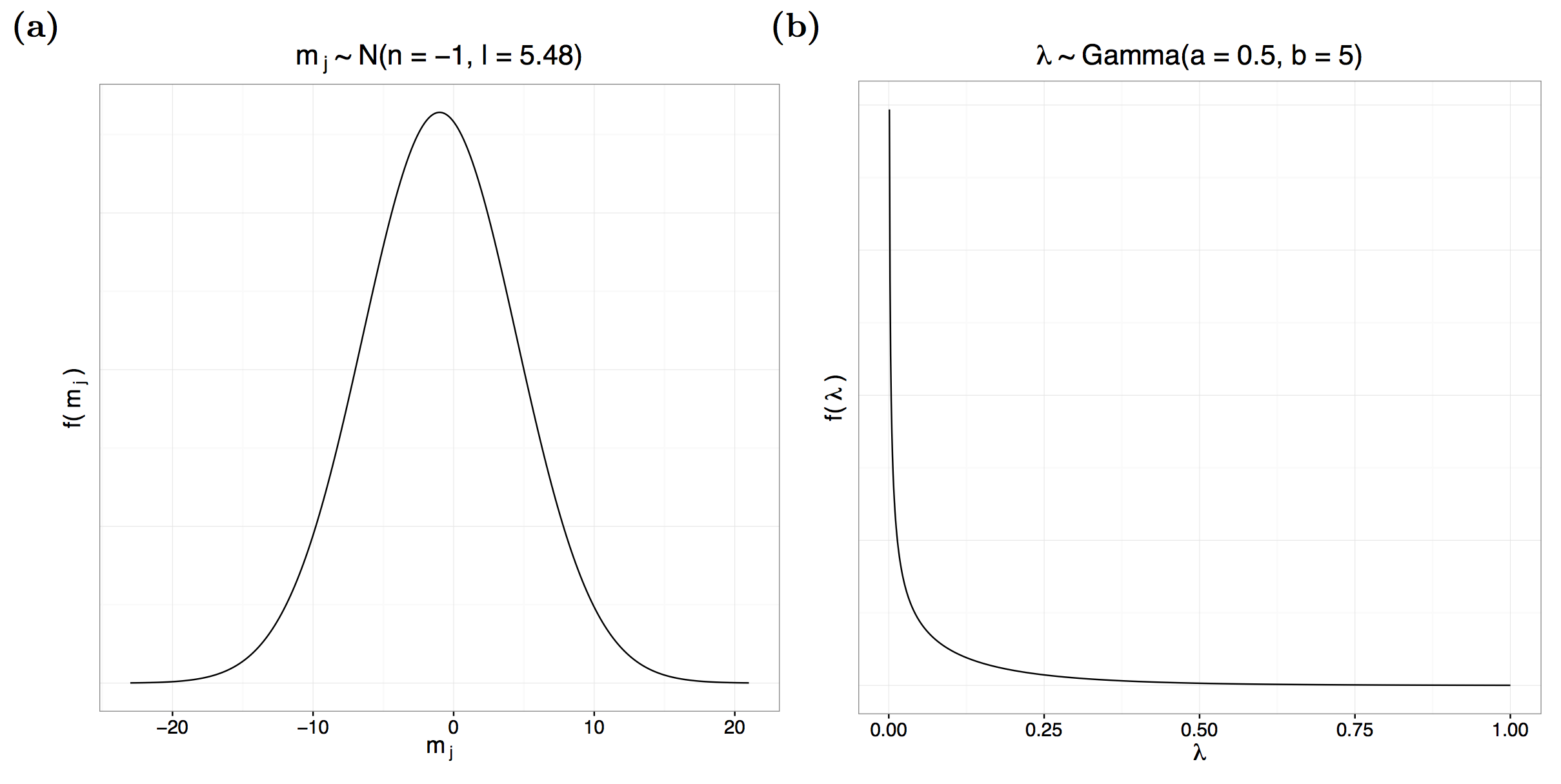

Supplement: S16 Fig — For our larger datasets simulated based on a stool dataset with 89 features, we used a prior for m that gave reasonable behavior for the sum of the basis medians ∑j=189emj (A). The prior on λ put most prior weight on λ values less than one and had narrow tails to encourage shrinkage of the correlation estimates (B). (See also S14 Fig). (TIF) [file pcbi.1005852.s022.tif]
